# Single-Cell Transcriptional Profiling and Gene Regulatory Network Modeling in Tg2576 Mice Reveal Gender-Dependent Molecular Features Preceding Alzheimer-Like Pathologies

**DOI:** 10.1007/s12035-022-02985-2

**Published:** 2022-08-18

**Authors:** Muhammad Ali, Oihane Uriarte Huarte, Tony Heurtaux, Pierre Garcia, Beatriz Pardo Rodriguez, Kamil Grzyb, Rashi Halder, Alexander Skupin, Manuel Buttini, Enrico Glaab

**Affiliations:** 1 Luxembourg Centre for Systems Biomedicine (LCSB), University of Luxembourg, 7 avenue des Hauts Fourneaux, L-4362 Esch-sur-Alzette, Luxembourg; 2 School for Mental Health and Neuroscience (MHeNs), Department of Psychiatry and Neuropsychology, Maastricht University, 6200 Maastricht, the Netherlands; 3 Luxembourg Center of Neuropathology (LCNP), L-3555 Dudelange, Luxembourg; 4 Department of Life Sciences and Medicine (DLSM), University of Luxembourg, L‑4362 Esch-Sur-Alzette, Luxembourg; 5 University of the Basque Country, Cell Biology and Histology Department, 48940 Leioa, Vizcaya, Basque Country Spain; 6 Department of Physics and Materials Science, University of Luxembourg, 162a av. de la Faïencerie, 1511 Luxembourg, Luxembourg; 7 Department of Neuroscience, University of California San Diego, 9500 Gilman Dr, La Jolla, CA 92093 USA

**Keywords:** Alzheimer’s disease, Bioinformatics, Tg2576, Single-cell transcriptomics, Meta-analysis, Pathway analysis, Network analysis, Sex differences, Gender differences

## Abstract

Alzheimer’s disease (AD) onset and progression is influenced by a complex interplay of several environmental and genetic factors, one of them gender. Pronounced gender differences have been observed both in the relative risk of developing AD and in clinical disease manifestations. A molecular level understanding of these gender disparities is still missing, but could provide important clues on cellular mechanisms modulating the disease and reveal new targets for gender-oriented disease-modifying precision therapies. We therefore present here a comprehensive single-cell analysis of disease-associated molecular gender differences in transcriptomics data from the neocortex, one of the brain regions most susceptible to AD, in one of the most widely used AD mouse models, the Tg2576 model. Cortical areas are also most commonly used in studies of post-mortem AD brains. To identify disease-linked molecular processes that occur before the onset of detectable neuropathology, we focused our analyses on an age with no detectable plaques and microgliosis. Cell-type specific alterations were investigated at the level of individual genes, pathways, and gene regulatory networks. The number of differentially expressed genes (DEGs) was not large enough to build context-specific gene regulatory networks for each individual cell type, and thus, we focused on the study of cell types with dominant changes and included analyses of changes across the combination of cell types. We observed significant disease-associated gender differences in cellular processes related to synapse organization and reactive oxygen species metabolism, and identified a limited set of transcription factors, including *Egr1* and *Klf6*, a*s* key regulators of many of the disease-associated and gender-dependent gene expression changes in the model. Overall, our analyses revealed significant cell-type specific gene expression changes in individual genes, pathways and sub-networks, including gender-specific and gender-dimorphic changes in both upstream transcription factors and their downstream targets, in the Tg2576 AD model before the onset of overt disease. This opens a window into molecular events that could determine gender-susceptibility to AD, and uncovers tractable target candidates for potential gender-specific precision medicine for AD.

**Supplementary Information:**

The online version contains supplementary material available at 10.1007/s12035-022-02985-2.

## Background

Alzheimer’s disease (AD) is the most prevalent age-related neurodegenerative disorder and is clinically characterized by loss of memory and cognitive function [1]. There are currently almost 50 million people worldwide living with this debilitating disease, and this number is expected to reach about 80 million in 2030. The total costs of AD are estimated at around $820 billion, and could reach up to $2 trillion in 2030 [2]. A small subset of AD cases (10%) are familial, but the vast majority are sporadic and late-onset [3].

In the incidence and clinical manifestations of AD, significant gender differences have been observed [4, 5]. Females are reported to have a higher disease incidence rate in the older age groups even after adjusting for differential survival [6], have a more global AD pathology than men [7], and the same degree of pathology is more likely to result in an AD diagnosis in females than males [8], even though studies indicate that males suffer a more aggressive disease progression and earlier mortality [5]. A molecular level understanding of these complex and multifaceted gender disparities is still missing, but could provide important clues on cellular mechanisms modulating the disease, and help pave the way toward a precision-medicine oriented disease-modifying therapy.

AD-related investigations in human tissue samples are limited by the fact that for the early, pre-symptomatic stages, only limited information can be obtained from peripheral tissue samples, and brain tissue samples are generally only available at the post-mortem stage. Murine models of AD provide a means to address these limitations, by enabling a direct investigation of AD-related pathological alterations in the brain at early disease stages.

While previous genome-scale molecular investigations of AD mouse models have focused on bulk analyses of biospecimens with heterogeneous compositions of cell types, cell-type specific alterations and differential changes in distinct cell populations, which are particularly relevant for heterogeneous brain tissues such as the neocortex, may be missed by these conventional analysis methods. Human studies have analysed post-mortem AD samples with substantial plaque and tangle pathologies [9, 10]. A recent single-cell cell gene expression profiling included early stage AD [9], but none so far has looked at, for obvious ethical reasons, preclinical AD. With the help of animal models, such approaches become accessible, and they can pinpoint molecular alterations that precede the appearance of functional neuronal decline and neuropathological features, such as plaques, tangles, cognitive decline, and neurodegeneration. We hypothesized that they could help uncover these types of alterations as they relate to gender susceptibility in AD.

Here, we therefore present an analysis of one of the most heterogeneous brain tissues affected in AD [11, 12], the neocortex, using single-cell RNA-seq analyses, on a widely used AD mouse model, the Tg2576 model [13]. Tg2576 mice show age-dependent appearance of beta amyloid (Abeta) plaques, and memory deficits [14, 15]. Importantly, this model displays more pronounced memory deficits in females starting at 12 months [16], making it a suitable model to study gender specific molecular alterations that may be linked to these functional deficits. To identify molecular changes that precede overt pathology, and thus could help shed light into why females are more susceptible to AD, we focused our analyses on mice that did not have AD-like pathology yet.

The manuscript is structured as follows: We first present transgene-associated transcriptomic gender differences identified in single genes, covering gender-neutral (significant in both genders), gender-specific changes (significant in only one gender, and not approaching significance in the other), and gender-dimorphic changes (significant in both genders, but with opposite direction of the change). Then, we investigate transgene-associated gender differences of cellular pathways and processes, and we build gene regulatory networks (GRNs) for the cell-type with the most pronounced gender-specific sub-network changes (male-specific alterations in endothelial cells), as well as for the combined set of gender-specific and gender-dimorphic differentially expressed genes across all cell-types, to identify their main upstream regulators. Finally, we present perturbation analyses of the AD-associated GRN on the Tg2576 model scRNA-seq data, which revealed two key transcription factors, *Egr1* and *Klf6*, that control many gender-related downstream expression changes. We discuss these regulatory genes, many of which have previously already been implicated in AD, in the context of prior knowledge from the literature, functional annotation databases, and public eQTL and mutation data. It emerged that the pharmacological modulation of *Egr1* activity has the potential to alleviate gender-dependent pathological alterations in Tg2576 mice, and thus possibly AD. Our study presents, for the first time, an exhaustive description of preclinical molecular events, many of them significantly gender-associated, that occur before the appearance of AD lesions, in the widely used Tg2576 mouse model for AD.

## Results

### Twenty-Four-Week-Old Tg2576 Mice Show no Gender differences in Total App or hAPP, and Have no Detectable Abeta Plaques nor Microglial Activation

We were interested in uncovering gender-linked molecular events that happen before the onset of frank AD-linked pathologies, since these could help open up ways for gender-specific precision targets that could be tackled in the earliest phases of the disease. Hence, we picked 24-week-old male and female heterozygous Tg2576 and age-matched wildtype (WT) littermates for our analyses.

First, we checked if there were gender differences in the levels of total APP and hAPP. Because of the high sequence identity between the human *APP* gene and the mouse *App* gene, our RNA-seq data (see below) could not distinguish between both, although it is possible that small sequence differences may still lead to human *APP* being less well picked up by the RNA-seq than mouse *App*. Overall, the RNA-seq data showed that there were no significant detectable gender-associated differences in *APP*/*App* in Tg2576 mice compared to WT mice. Considering both genders together, we observe a nominally significant increased expression of *App*/*APP* in TG mice compared to WT controls in neurons (*p* value = 1.72E-05, adjusted *p* value = 0.33) using a Poisson generalized linear model [17]. To further assess the relevance of this expression difference, we applied another alternative differential expression analysis method (MAST approach [18]) and observed a significant increase in *App*/*APP* expression in TG mice also after adjustment for multiple hypothesis testing (*p* value = 4.25E-07, adjusted *p* value = 0.0083). We also measured average pixel intensity in the cortex of WT and Tg2576 mice of both genders, using fluorescently-labeled sections for total APP and hAPP, further confirming that the Tg2576 mice carry the transgene (Supplementary Fig. S1). We did not detect gender differences in either genotype for these measurements. This is in line with the reported absence of gender differences in total Abeta levels and in amyloid deposition in these mice [16]. Thus, Tg2576 mice are a suitable model to study molecular gender differences driven by hAPP/Abeta.

For the 24-week-old mice, by immunohistochemistry, we did not detect any Abeta deposits in the brains of these mice, nor did we find evidence for microglial activation (Supplementary Fig. S2). Published evidence indicates that, at that age, Tg2576 mice memory performance is slightly impaired in the Morris Water maze [15], but not impaired in the Radial Arm Water Maze [14]. Female Tg2576 mice have worse memory performance than their male counterparts in a Hole Board test starting at 12 months of age, but not at earlier ages [16]. In these studies, correlations between the amount of insoluble Abeta and/or Abeta plaques, but not transgenic hAPP levels, indicated that the memory and cognition impairment were caused by Abeta and not the transgene, stressing the AD relevance of the Tg2576 model. Thus, choosing 24-week-old mice seemed adequate to capture early, gender-specific Abeta-driven molecular changes in this model.

### Clustering of Single-Cell RNA-seq Data and Cell Type Identification

**Fig. 1:**
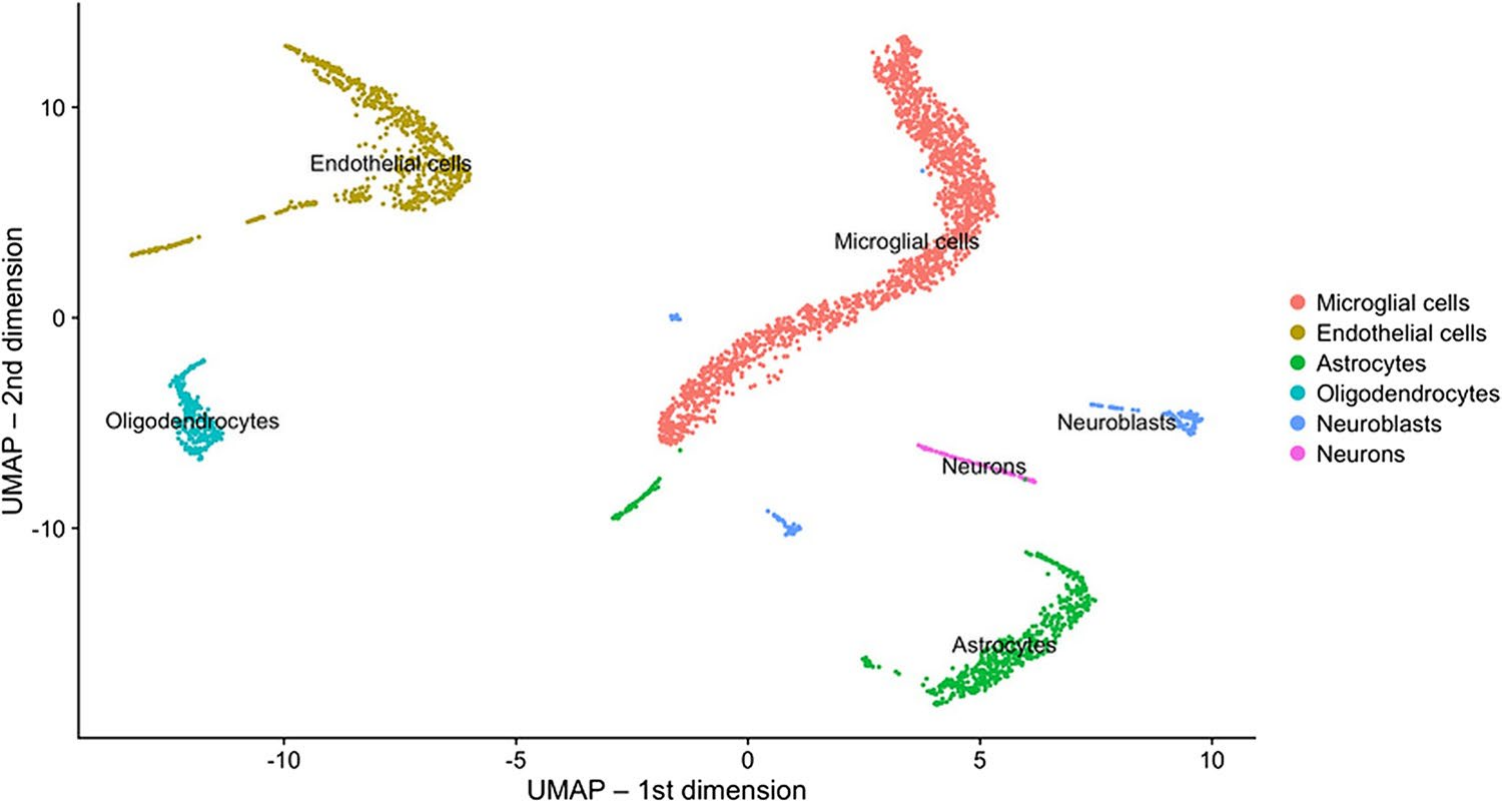
Two-dimensional cluster visualization of the single-cell RNA-seq data with added cell type annotations (using data from all mice across both genders). The visualization was generated using the Uniform Manifold Approximation and Projection (UMAP) approach for dimension reduction

We conducted single-cell RNA-seq profiling analyses using the DropSeq approach on mouse neocortical samples from 24-week-old Tg2576 mice and WT littermate control mice for both genders. After pre-filtering and pre-processing the raw read count data (see “Methods”), we first determined cell-type clusters using the shared nearest neighbor (SNN)-Cliq approach, a graph-based clustering method which groups cells together depending on their shared or distinct expression patterns [19]. The optimal number of clusters was determined by scoring the results for different possible settings using a cluster validity index, the Silhouette Width [20], which provided the maximum score (0.5) for 6 clusters (see “Methods”). This approach identified six distinct clusters of cell types using the measured single-cell gene expression data from all mouse samples. A corresponding 2D visualization of these clusters, including their cell-type annotations determined as described in the following, is shown in Fig. 1 (created using the UMAP approach [21], Supplementary Fig. S3 shows corresponding genotype- and gender-specific cluster visualizations).

To obtain unique cell type annotations for each cluster, cell-type specific marker genes with pronounced expression in each cluster were determined using the Cell Marker database [22]. The largest cluster, annotated for microglial cells, exhibited high expression levels of the genes *P2ry12*, *Hexb*, and *Ctss*, which have all previously been reported as homeostatic microglial cell markers [23, 24]. Similarly, a second prominent cluster which expressed known oligodendrocyte markers, such as *Cldn11* and *Ermn* [24], was annotated for this cell type.

**Fig. 2:**
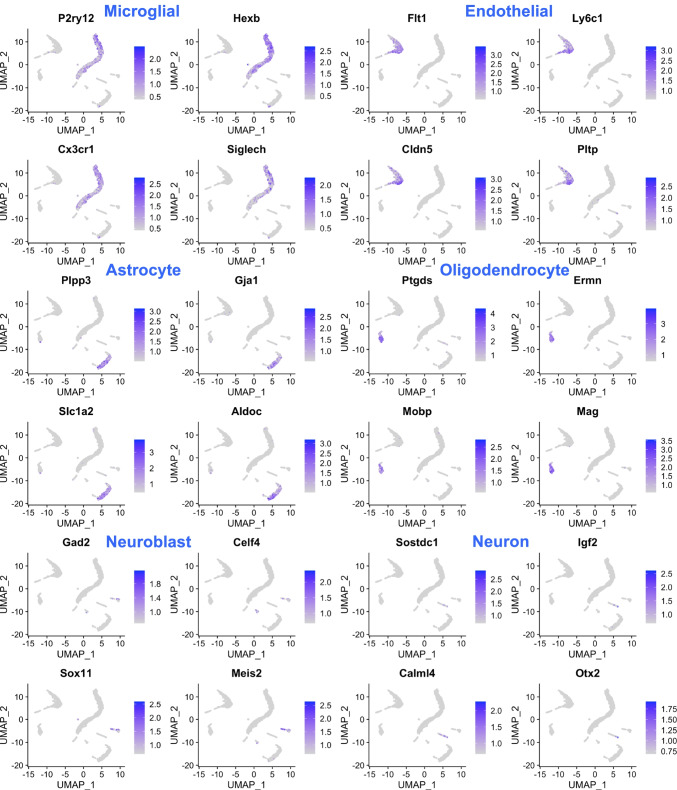
Visualization of marker gene expression across different clusters. The cluster cell type annotations are shown in blue color at the top of the 2D cluster visualizations, which highlight the marker genes that show a pronounced expression in certain cell-type specific clusters. Marker gene symbol labels are indicated in black above the plots

Apart from microglial cells (1671 cells) and oligodendrocytes (255 cells), other cell type clusters included endothelial cells (618 cells), astrocytes (575 cells), neuroblasts (137 cells), and neurons (81 cells). The corresponding cluster annotations and their associated marker genes are shown in Suppl. Table 1. Figure 2 highlights the expression of key marker genes across different cell type clusters.

The method we used included a myelin removal step, which leads to substantial loss of neurons [25]. While neuronal demise is at the base of AD-linked cognitive decline and, ultimately, dementia, the role of other CNS cells in promoting, or being harmed by the disease process cannot be understated. Microglia, which represent the dominant cell type in our data, are a major driver for AD pathologies [26–28], and a number of microglial gene variants are linked to increased risk for late-onset AD [29]. Endothelial cells of the brain vasculature are susceptible to Abeta toxicity, can have Abeta deposits [30], and their dysfunction is believed to contribute to AD pathology [31]. Astrocytes respond to Abeta, become reactive in AD, and their dysfunction is believed to contribute to neuroinflammation and disruption of synaptic function [32]. Finally, oligodendrocytes are particularly susceptible to insults such as neuroinflammation [33], myelin degeneration has been reported in AD [34], and gene expression changes in these cells are a recently uncovered feature of the disease [9]. Hence, the method for cell isolation we used opened up a window into the molecular events in several AD-relevant cell populations before the appearance of frank AD pathologies.

### Gene-Level Analysis of AD-Associated Molecular Gender Differences

**Fig. 3:**
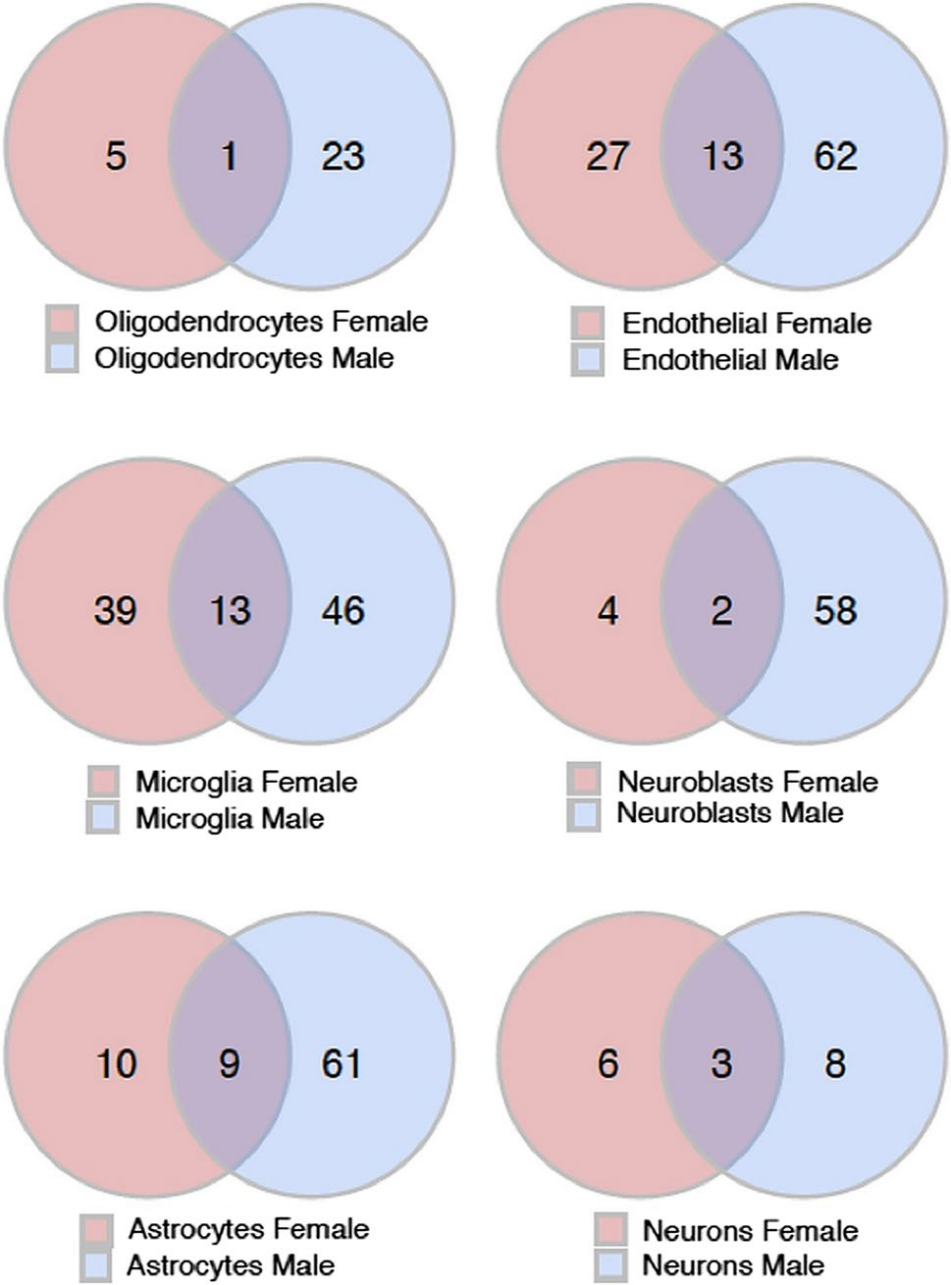
Venn diagram visualization of the number of significant DEGs between Tg2576 mice and WT controls that are gender-neutral (i.e., overlapping between genders and in the intersection set of the Venn diagram) and those that are gender-specific (non-overlapping) across different cell type clusters. Male and female groups are represented by blue and red circles, respectively. The numbers reflect the number of significant DEGs (Q value < 0.05) between the genotypes in the intersecting or non-intersecting male and female sample groups (e.g., in oligodendrocytes 5 DEGs between TG and WT mice were specific to females, 23 were specific to males, and 1 DEG was shared across the genders; for the other cell types, DEGs were split in the same manner)

We determined significant gender-neutral, gender-specific, and gender-dimorphic differentially expressed genes (DEGs) between the genotypes in both a cell type specific and cell type agnostic manner, using a Poisson generalized linear model [17]. Gender-neutral DEGs were defined as the genes with shared direction of change (shared sign of the log. fold change) and a Bonferroni-adjusted *p* value (*q* value) < 0.05 in both genders of Tg2576 mice. Gender-specific DEGs were defined as displaying significant differences in only one gender (*q* value < 0.05), while not approaching significance in the other gender (*q* value > 0.5; the higher *q* value was applied to exclude spurious findings of genes that could be interpreted as gender-specific, with a significance close to the common *q* value threshold of 0.05). Finally, gender-dimorphic DEGs were defined as genes that are significantly altered in both genders (*q* value < 0.05), but with opposite directionality, i.e., up in one gender, and down in the other (with different signs of the log. fold changes). Detailed lists of the top 10 identified gender-neutral, gender-specific, and gender-dimorphic DEGs are shown in Tables 1, 2, and 3. A Venn diagram visualizing the size of the overlap between significantly DEGs in different cell-type specific clusters for both genders, as well as the male- and female-specific DEGs, is shown in Fig. 3, and a heat map of the expression levels for the genes with significant AD-associated gender differences across the combination of cell types is displayed in Supplementary Fig. 4.

**Table 1:**
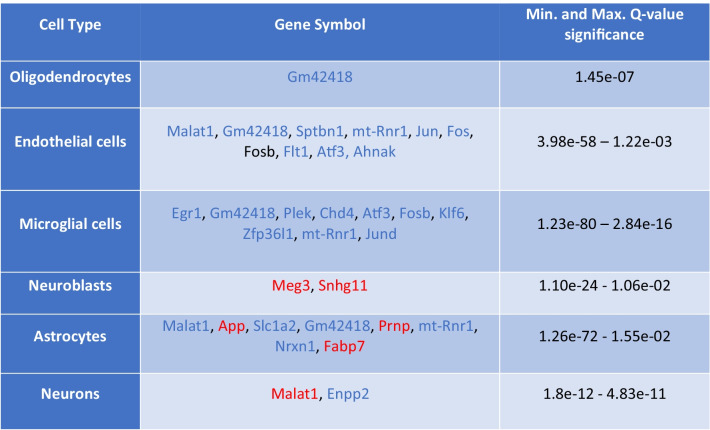
Gender-neutral DEGs

Table of the most significant gender-neutral DEGs (*q* value < 0.05, up to 10 genes per cell type shown), i.e., the shared differentially expressed genes in both genders (male and female) across different cell types in Tg2576 mice compared to WT controls. Genes with increased versus decreased expression (compared to WT controls) are represented by red and blue color, respectively. The complete lists of DEGs across all cell types, including the logFC and *q* value statistics for each gene in both genders, are provided in Supplementary Table S2

**Table 2:**
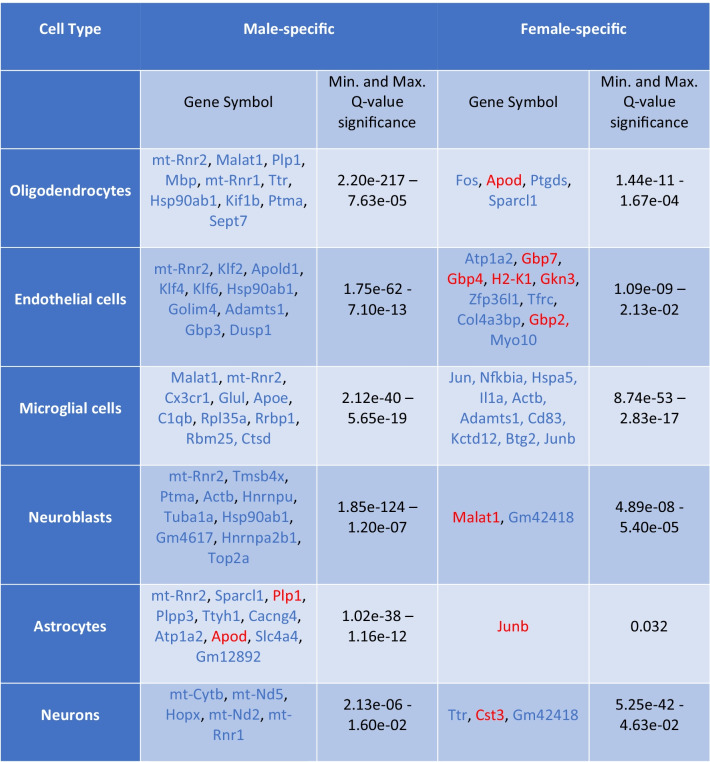
Gender-specific DEGs

Table of the most significant gender-specific DEGs (*Q* value < 0.05, up to 10 genes per cell type shown), i.e., the genes which are differentially expressed in only one gender (male or female, *Q* value < 0.05), and which do not approach significance in the other gender (*Q* value > 0.5), in specific cell types in Tg2576 mice compared to WT controls. Genes with increased versus decreased expression (compared to WT controls) are represented by red and blue color, respectively. The complete lists of gender-specific DEGs across all cell types are provided in the Supplementary Table S2

**Table 3:**
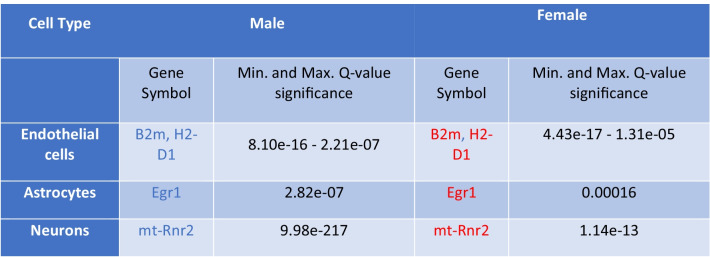
Gender-dimorphic DEGs

Table of the most significant gender-dimorphic DEGs (*Q* value < 0.05), i.e., significantly differentially expressed in both genders (male and female) but with an opposite log. fold-change per cell type, in Tg2576 mice compared to WT controls. Genes with increased or decreased expression compared to WT controls are represented by red and blue color, respectively. The complete lists of DEGs across all cell types are provided in the Supplementary Table S2

Overall, more male-specific than female-specific DEGs were identified in all cell types (see Table 2 and the Supplementary Tables S1 and S2 for the DEGs per cell type and related statistics). In the analysis of gender-specific DEGs across the combination of all cell types, 22 male-specific and 5 female-specific DEGs were identified, most of them with reduced expression in Tg2576 mice compared to WT controls.

The largest numbers of significant DEGs were detected in endothelial cells (60 in total) and microglial cells (52), which may reflect the key involvement of endothelial cells in vascular AD and the central role of microglia in AD progression (see the Discussion of gender-neutral genes, among which the largest number of DEGs was found in microglial cells). In astrocytes, two gender-neutral DEGs were of note: enhanced expression of murine/human *App*/*APP* and reduced expression of *Slc1a2* (coding for glutamate transporter 1). Astrocytes have been suggested as a source of APP-derived Abeta in this model, thus contributing to AD pathology [35]. The increased production of App in this model may be part of their response to hAPP/Abeta. On the other hand, through glutamate reuptake via the glutamate transporter, they may limit the extracellular presence of excitotoxic glutamate [36]. A reduced re-uptake of excitotoxic glutamate may contribute to neuronal dysfunction in this model.

The total number of DEGs identified does not necessarily provide an indicator of disease severity, because DEGs can reflect both pathological changes and protective responses (see the discussion of the specific cellular processes enriched in DEGs and their gender associations in the section on “Pathway-level analysis of AD-associated molecular gender differences”).

**Fig. 4:**
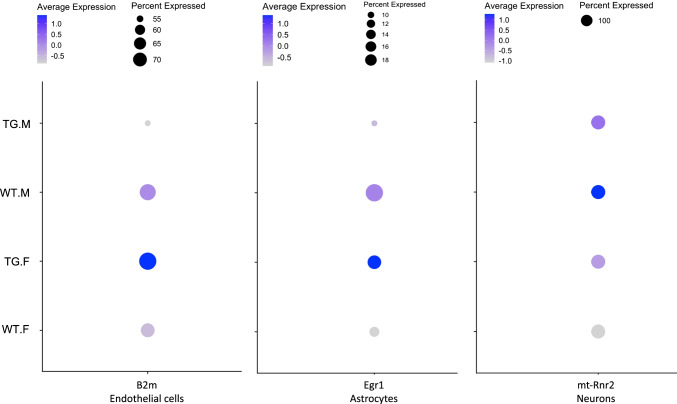
Dot plots of the normalized expression levels of the gender-dimorphic genes B2m (left), Egr1 (center), and mt-Rnr2 (right) in endothelial cells, astrocytes, and neurons, respectively. Both average expression (higher to lower average expression is represented by a blue to grey color gradient) and the percentage of cells expressing the relevant gene (reflected by the size of the dots, see legend on the right) are displayed. For B2m, a decrease in the percentage of endothelial cells expressing the gene and lower average expression was observed in Tg2576 males (TG.M) compared to WT males (WT.M), and higher average expression observed in Tg2576 females (TG.F) compared to WT females (WT.F). For Egr1, a decrease in the percentage of cells expressing the gene and lower average expression was found in Tg2576 males compared to Tg2576 females, and an increase in the percentage of cells expressing the gene and higher average expression in Tg2576 females compared to WT females. For mt-Rnr2, higher average expression was observed in Tg2576 females (TG.F) compared to WT females (WT.F), while the opposite patterns was seen in males (no significant change in the percentage of cells expressing the gene was observed)

Among the most pronounced gender-dimorphic alterations, significant expression changes were observed in the gene for beta-2-microglobulin (*B2m*) in endothelial cells. *B2m* displayed significant alterations with adjusted *p* values (*q* values) of 4.4e-17 (female) and 8.1e-16 (male), and log. fold changes (logFCs) with opposite direction of 0.68 (female) and − 0.43 (male, see also the dot plot in Fig. 4, left). B2m, a component of MHC class 1, has been reported to be elevated in serum of AD patients [37] and in endothelial cells of human AD [38]. As a regulator of synaptic plasticity, it was shown to impair cognitive function and neurogenesis in mice [39]. We note that in Tg2576 mice at the studied age, alterations in synaptic spine density are among the most obvious changes to detect, as the decrease in spine density in the outer molecular layer of the dentate gyrus (DG) was described to begin as early as 4 months of age [40]. Moreover, B2m and the major histocompatibility complex (MHC) Class 1 molecule H2-D1, which displayed gender-dimorphic changes with the same directionality as B2m in endothelial cells (see Table 1), are directly interacting in the murine MHC complex H-2Db and have been shown to be upregulated with age, more so in female than in male mice [41].

Another significant gender-dimorphic change was observed in astrocytes for the gene *Egr1* (Early growth response-1), with logFCs of 0.53 (males) and − 0.31 (females), and *q* values of 1.6e-4 and 2.82e-07, respectively (see dot plot in Fig. 4, center). *Egr1*, which belongs to the “immediate early gene” family of transcription factors, is known to modulate AD pathologies in model organisms [42, 43]. Interestingly, its inhibition has been reported to ameliorate AD-like phenotypes in the 3xTg mouse model of AD [44]. In the Tg2576 mice, our analysis showed that, across the totality of cell-types, *Egr1* is significantly decreased in both genders compared to controls (logFC in females: − 0.43, logFC in males: − 0.41, with *q* values of 1.17e-65 and 1.76e-100, respectively). Moreover, significant gender-related differences in *Egr1* expression also emerged when looking at different cell types individually. While in microglia *Egr1* expression differences followed the pattern observed across all cells (lower expression in Tg2576 mice compared to wildtype in both genders; logFC in females: − 0.54, logFC in males: − 0.36, with *q* values of 1.23e-80 and 3.65e-52, respectively), a significant gender-dimorphic change was observed in astrocytes (logFC in males: − 0.31, *q* value: 2.82e-07; logFC in females: 0.53, *q* value: 0.00016), and a male-specific decrease was observed in endothelial cells (logFC: − 0.26, *q* value: 0.0012).

In neurons, the only gene showing significant gender-dimorphic alterations was *mt-Rnr2* (Mitochondrially Encoded 16S RRNA, also called *Humanin*), with a decreased expression in males (logFC: − 1.08, *q* value: 9.98e-13) and an increased one in females (logFC: 0.83; *q* value: 1.15e-13). *mt-Rnr2* is an RNA gene with neuroprotective functions, suppressing apoptosis by binding to the protein Bax and blocking Bax translocation from the cytosol to mitochondria [45]. In the context of AD, *mt-Rnr2* has been reported to reduce aggregation and fibrillary formation of the 42-aa form of Abeta (Abeta42) by suppressing the effect of Abeta42 on mononuclear phagocytes and competitively inhibiting the access of FPRL1 to Abeta42 [46]. In in vitro and animal models of multiple neurodegenerative disorders, including AD, stroke and Huntington’s disease, *mt-Rnr2*, and derivated peptides displayed cytoprotective activity, by promoting increased survival and protecting against oxidative stress [47–51].

In summary, in our analyses, several statistically significant gender-associated DEGs were identified. Gender-specific expression changes were observed for each cell-type, and gender-dimorphic expression changes for two genes in endothelial cells (*B2m* and *H2-D1*), one gene in astrocytes (*Egr1*), and one gene in neurons (*mt-Rnr2*). Additional functional annotation descriptions for the significant male- and female-specific and gender-neutral DEGs not discussed in the main manuscript are provided in Suppl. Table 2.

### Pathway-Level Analysis of AD-Associated Molecular Gender Differences

To investigate coordinated alterations in cellular processes in Tg2576 mice and how they relate to gender, we characterized gender-neutral and gender-specific activity changes in biological pathways and processes by applying gene set enrichment analyses, using the databases Gene Ontology (GO) [52] and KEGG [53] (see Methods and Supplementary Figures S5-S9). These analyses were first conducted for all cell types combined (global analysis), then separately for each cell type (cell-type specific analysis). Because the expression changes in many pathways in different cell types pointed in opposite directions, they canceled each other out in the global analysis, and the resulting number of total enriched pathways in Tg2576 mice in the global analysis was lower compared to the results for some of the individual cell types. The large numbers of enriched pathways identified for individual cell types precludes a comprehensive discussion of all statistically significant findings. However, the detailed results for the cell-type specific enrichment analyses are only shown in Supplementary Tab. S3, providing an important resource for further research in the field. In the following section, we focus on gender-associated global pathway changes, and only discuss the most robust findings obtained from the global analysis across all cell types.

As input for this pathway analysis, the global scoring of differentially expressed genes identified 22 male-specific DEGs, 5 female-specific DEGs, and 15 gender-neutral DEGs, but no gender-dimorphic DEGs. Thus, for a robust enrichment analysis, the number of input DEGs was only large enough for the male-specific and the gender-neutral DEGs, and we therefore discuss only pathways enriched in these two groups of DEGs.

#### Pathways Enriched in Gender-Neutral DEGs

**Fig. 5:**
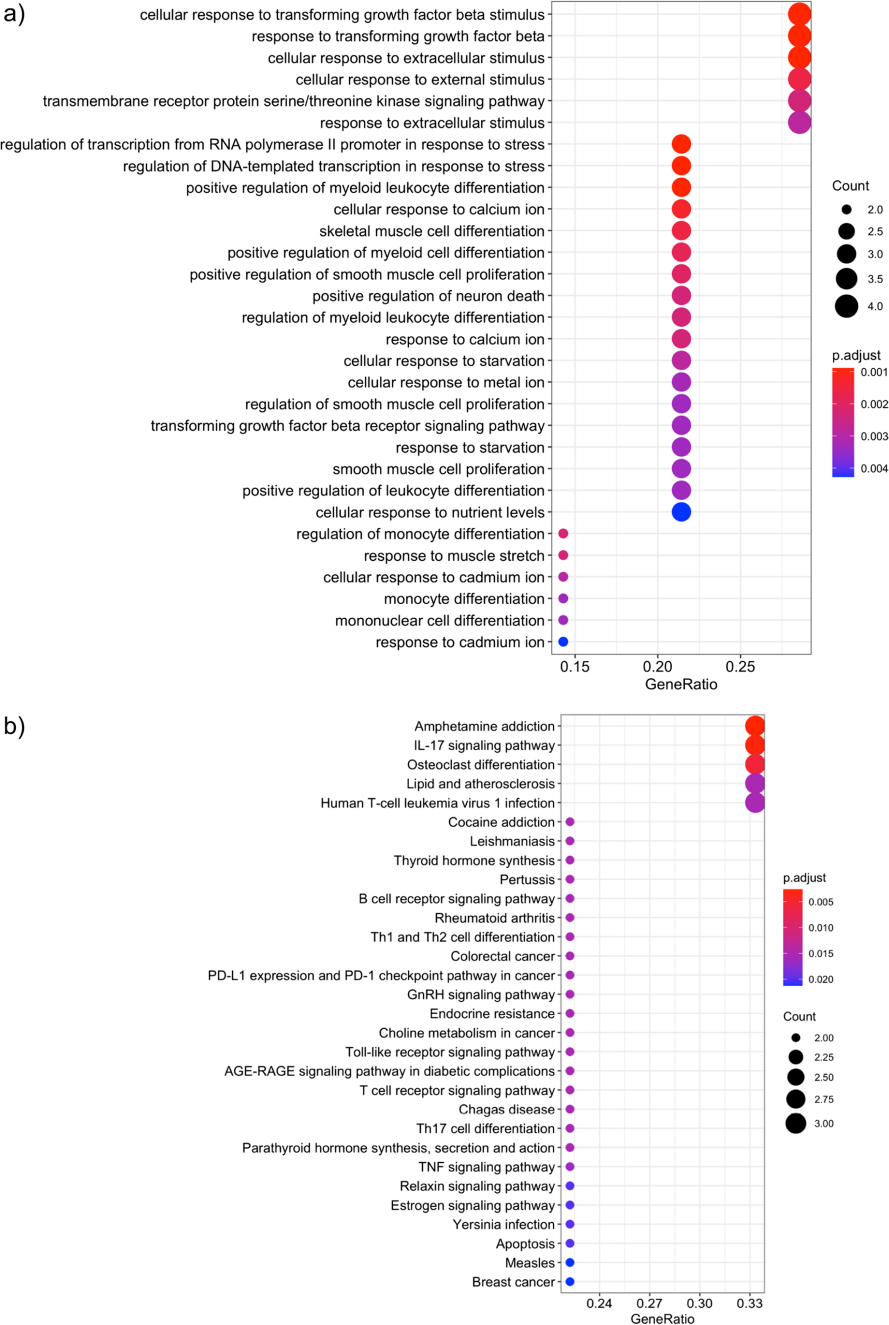
Visualization of the top biological processes and pathways with an over-representation of Tg2576-associated gender-neutral DEGs, extracted from the pathway members for Gene Ontology biological processes (**a**), and the KEGG database (**b**). The size of the circle represents the number of genes associated to a particular process or pathway, and the color represents the significance level. The horizontal axis shows the gene ratio, i.e., the number of DEGs assigned to a particular biological process or pathway in relation to the size of the overlap with all members of all covered pathways (larger ratios indicate a stronger over-representation; see legend on the right)

As a main observation, the pathways with a significant over-representation of gender-neutral DEGs (*q* value < 0.05) include many cellular stress response processes among the enriched GO terms. These cover processes such as “cellular response to starvation” (GO:0,009,267), “cellular response to metal ion” (GO:0,071,248), “regulation of DNA-templated transcription in response to stress” (GO:0,043,620), and “regulation of transcription from RNA polymerase II promoter in response to stress” (GO:0,036,003). Additionally, multiple TGF beta signaling related pathways (e.g., “cellular response to transforming growth factor beta stimulus,” “response to transforming growth factor beta,” and “transforming growth factor beta signaling”), immune and inflammatory response pathways (“IL-17 signaling pathway,” “Th1 and Th2 cell differentiation,” “T cell receptor signaling pathway,” “Toll-like receptor signaling pathway,” and “Th17 cell differentiation”), and cell death related pathways (“positive regulation of neuron death,” “Apoptosis”) showed a significant enrichment. An overview of the top 30 enriched biological processes and pathways from the Gene Ontology and KEGG databases with a significant over-representation of gender-related differentially expressed genes is shown in Fig. 5. Comprehensive pathway ranking tables with additional enrichment statistics for the individual cell types are provided in Supplementary Table S3.

#### Pathways Enriched in Male-Specific DEGs

**Fig. 6:**
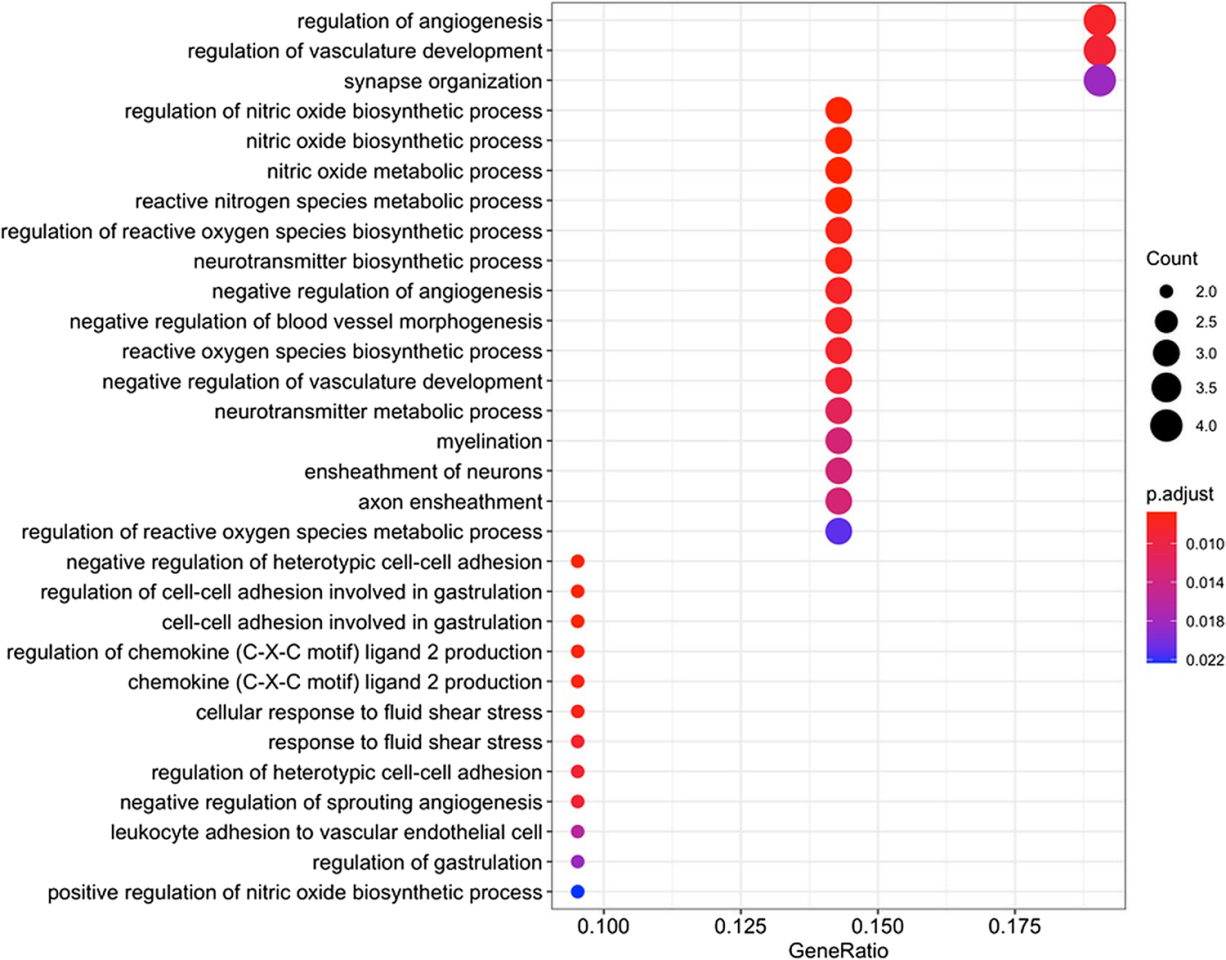
Visualization of the top biological processes and pathways with an over-representation of Tg2576-associated male-specific DEGs among the pathway members for Gene Ontology biological processes (BP). The size of the circle represents the number of genes associated to a particular process or pathway, and the color represents the significance level. The horizontal axis shows the gene ratio, i.e., the number of DEGs assigned to a particular biological process or pathway in relation to the size of the overlap with all members of all pathways (larger ratios indicate a stronger over-representation, see legend on the right)

Among the cellular processes with an over-representation of male-specific DEGs, many were associated with angiogenesis and vasculature development, including “regulation of angiogenesis” (GO:0,045,765), “regulation of vasculature development” (GO:0,001,944), “negative regulation of angiogenesis” (GO:0,016,525), “negative regulation of sprouting angiogenesis” (GO:1,905,554), and “negative regulation of vasculature development” (GO:0,016,525, see Fig. 6). Furthermore, male-specific changes were enriched in nitric oxide and nitrogen species metabolic processes (“regulation of nitric oxide biosynthetic process,” “nitric oxide biosynthetic process,” “nitric oxide metabolic process,” “reactive nitrogen species metabolic process,” and “positive regulation of nitric oxide biosynthetic process”), and general reactive oxygen species metabolic processes (“regulation of reactive oxygen species biosynthetic process,” “reactive oxygen species biosynthetic process,” and “regulation of reactive oxygen species metabolic process”). Finally, neurotransmitter related processes (“neurotransmitter biosynthetic process,” “neurotransmitter metabolic process”), cell adhesion processes (“negative regulation of heterotypic cell–cell adhesion,” “regulation of heterotypic cell–cell adhesion”), and myelination/ensheathment processes (“myelination,” “ensheathment of neurons,” and “axon ensheathment”) displayed significant male-specific alterations. Figure 6 provides a more detailed overview of the top 30 significant Gene Ontology biological processes with male-specific DEG enrichment (no significant associations were identified for the smaller KEGG database). A complete list of all significant pathways, including information on the overlapping genes and detailed statistics is provided in the Supplementary Table S3.

### Regulatory Network Analysis of AD-Associated Molecular Gender Differences

Since systems-level molecular changes in omics data cannot always be captured by classical pathway analyses, we next performed a differential gene regulatory network (GRN) analysis on the Tg2576 model data. This allowed us to determine key regulatory genes predicted to be involved in driving and maintaining the disease phenotype. Specifically, we applied a previously developed differential GRN inference approach [54] separately to the male- and female-specific DEGs obtained for individual cell types and for the totality of all cells. Sufficient DEGs for robust network construction were only obtained for the more numerous male-specific DEGs and only for few cell types, or in combined analyses across all cell types. Therefore, in the following paragraphs, we focus on the discussion of the two most robust sub-network alterations identified: A sub-network with significant male-specific DEGs in endothelial cells, and a sub-network of male-specific DEGs across the combination cell types.

#### Endothelial Sub-Network with Male-Specific DEGs

**Fig. 7:**
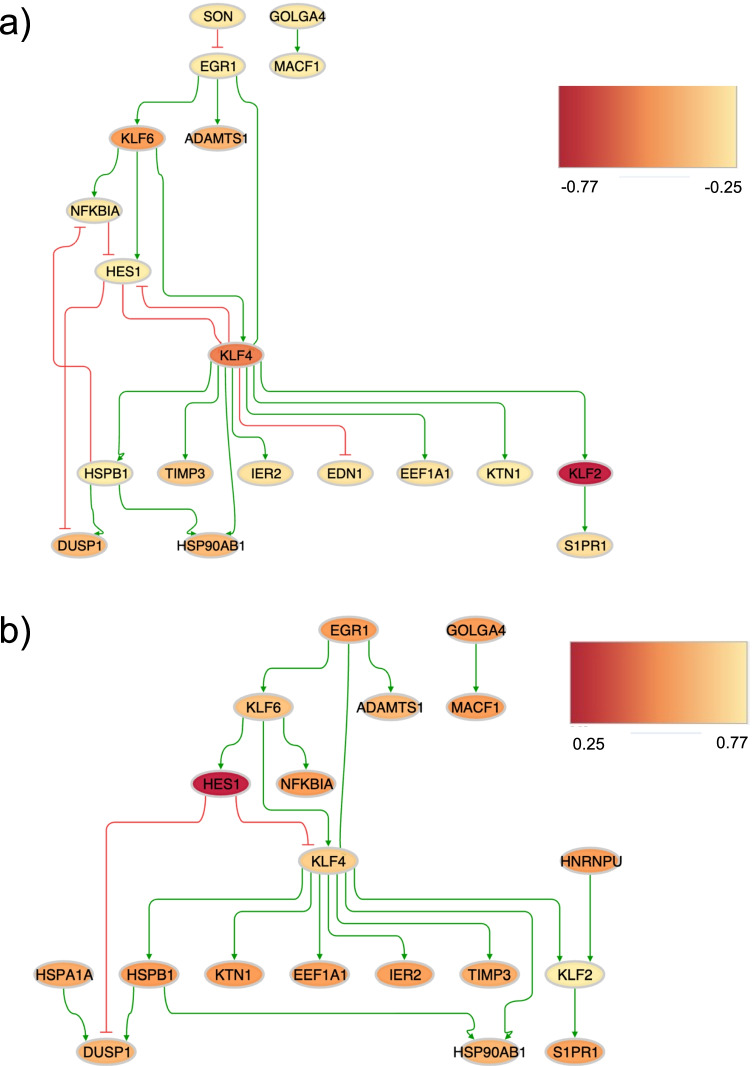
Visualization of the GRNs for the two genotypes, Tg2576 (**a**) and WT (**b**), enriched in male-specific DEGs in endothelial cells. The networks highlight key regulatory transcription factors and their interactions with direct downstream target genes. The Tg2576 network (**a**) contains 19 genes and 24 interactions, and the WT network (**b**) covers 19 genes and 21 interactions. Green and red lines represent activating and inhibiting interactions, respectively. The genes (represented by nodes) are colored based on their absolute log fold changes (logFC) from most under-expressed (red color) to the most over-expressed (yellow)

When using the 47 male-specific DEGs identified in endothelial cells as input for the GRN construction, two sub-networks were obtained, representing the main regulatory networks controlling the male-specific expression in the Tg2576 mice and the WT control mice, respectively. The Tg2576-specific network covered 19 genes and 24 regulatory interactions (see Fig. 7a), and the WT network covered 19 genes and 21 interactions (see Fig. 7b). Among the transcription factors in the upstream region of the network (top part in Fig. 7 a and b), *Egr1*, *Klf4*, and *Klf6* did not only show a shared significant male-specific decreased expression in Tg2576 vs. WT in endothelial cells, but were also involved in significant transgene-associated alterations in other cell types (*Egr1* displayed gender-dimorphic expression alterations in astrocytes, *Klf4* a male-specific decrease in microglial cells, *Egr1* and *Klf6* a gender-shared decrease in microglial cells, and *Klf6* a male-specific decrease in neuroblast cells; see sheets 1 to 3 and Supplementary Table S2).

#### Sub-Network with Male-Specific DEGs Across all Cell Types

**Fig. 8:**
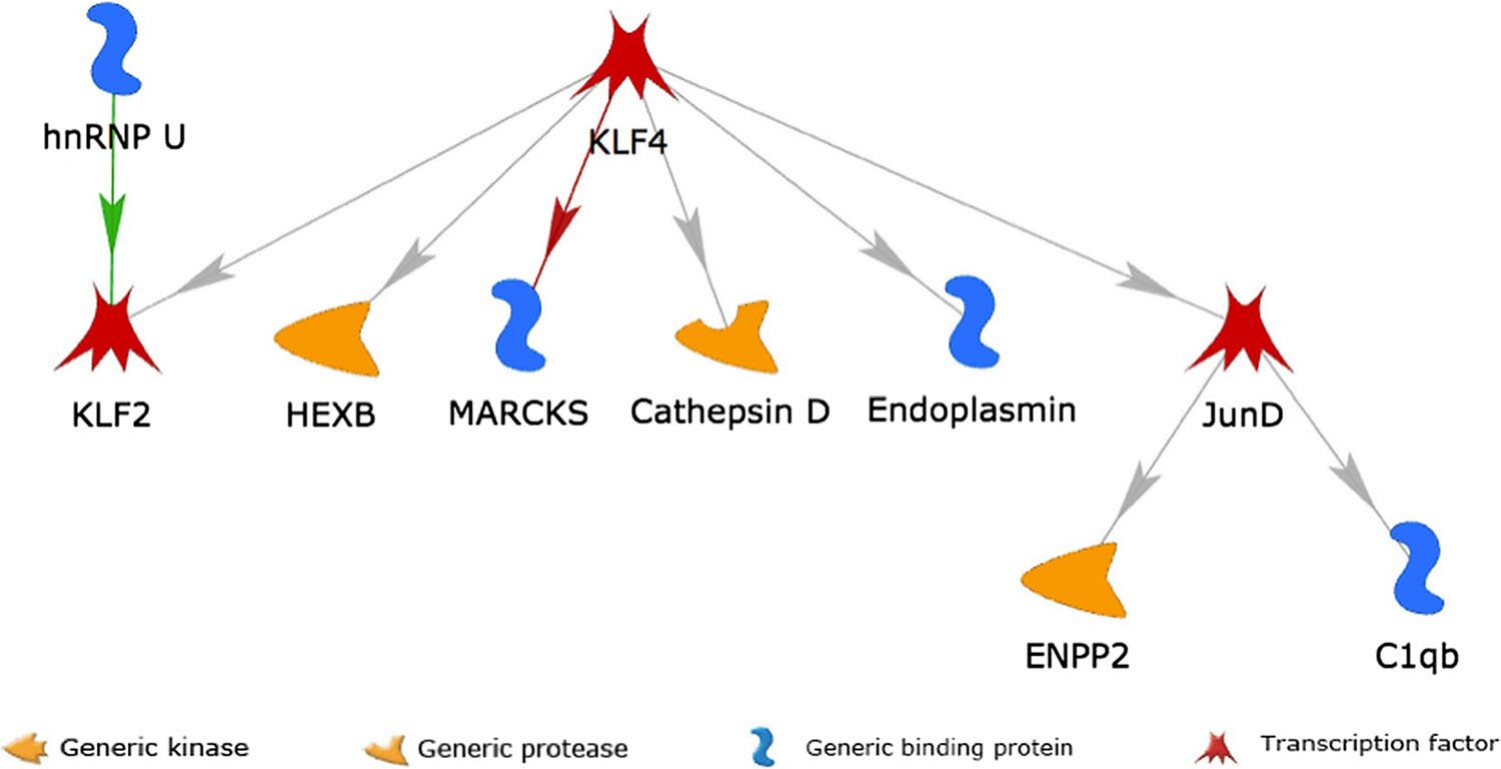
Visualization of the GRN interconnecting male-specific DEGs from all cell types combined. All genes in the network showed significantly lower expression in Tg2576 males than in WT littermate controls. Green and red lines represent activating and inhibiting interactions, respectively. Gray lines stand for interactions with unknown effect according to the prior literature (i.e., activating or inhibiting activity is both possible), but an activating effect is most likely due to the shared reduced expression of the upstream genes (Klf4, Jund) and their downstream genes (Klf2, Hexb, Marcks, Ctsd, Hsp90b1 (= Endoplasmin), and respectively, Enpp2 and C1qb). The types of proteins encoded by the genes are highlighted by the symbols annotated in the legend at the bottom. Due to the limited number of genes and interactions with annotated effects, no genotype-specific networks could be generated, and no upstream perturbation effects could be estimated for this network

In the global analysis of gender-specific and gender-dimorphic DEGs across all cell types, only 22 significant male-specific DEGs, 5 female-specific DEGs, but no gender-dimorphic DEGs were identified. With these available numbers of DEGs, only a male-specific GRN covering 10 male-specific DEGs and 9 interactions between them could be built (see Fig. 8), but no genotype-specific GRNs. All the genes in this sub-network had significantly lower expression in Tg2576 males than their WT littermates across the combined cell types. The transcription factor *Klf4* regulates most of the genes in this sub-network, either directly, or indirectly via its target transcription factor *Jund*.

For most interactions, no effect annotations were available (gray colored lines), i.e., prior knowledge on whether the target is activated or inhibited is missing. Given the matching expression changes between upstream transcription factors and their direct downstream targets, most of these interactions likely have an activating rather than an inhibiting effect.

Overall, the key role of the transcription factor *Klf4* in controlling a male-specific downregulation of several target genes across all cell types combined matches with the observations for the GRN built for endothelial cells, where *Klf4* also controlled several male-specific DEGs with reduced expression, and the significant male-specific reduced expression of *Klf4* in microglial cells.

To explore whether the key transcription factors identified in the two network analyses, *Egr1*, *Klf6*, and *Klf4*, or other regulatory genes from the GRNs may serve as candidate targets for follow-up preclinical pharmacological inhibition studies to reverse or alleviate gender-associated pathological alterations in AD, we complemented the gene regulatory network analysis by further network perturbation analyses (see following section).

### Network Perturbation Analysis to Identify Key Upstream Regulators of the AD Transcriptomic Phenotype in Tg2576 Mice

Recent studies suggest that modeling and interpreting complex diseases as molecular network perturbations provides a means to capture the multifaceted and diverse pathological alterations that trigger cascades of downstream disease-causing molecular events [55]. We therefore conducted in-silico network perturbation analyses (see “Methods” and [54]) on the sub-network with the most robust gender-specific changes, the GRN built for the endothelial male-specific DEGs, to determine key regulatory genes which control the expression of downstream genes with expression associated with the AD-phenotype in the male Tg2576 model.

While previous studies on sex differences in endothelial function are conflicting, with some studies showing similar age-related decline across the sexes, the studies that observed significant differences consistently showed earlier functional decrements in men than women, potentially due to a lack of cardioprotective estrogens [56]. Although the incidence of AD is higher for women than for men after the age of 90, even when adjusting for differential survival, the overall incidence of vascular dementia is lower in women compared to men (rate ratio 0.57, 95% CI: 0.34–0.97) [57]. Since endothelial dysfunction is thought to contribute significantly to vascular forms of AD [58], the regulatory genes controlling the transgene-associated changes in the Tg2576 endothelial network may provide candidate drug targets with the potential to pharmacologically revert gender-associated pathological changes in the Tg2576 disease phenotype.

Applying the perturbation analysis to this network provided scores for all candidate regulatory genes in the network (see Table 4), which reflect the total number of downstream genes whose disease-associated expression alterations can be reverted through the perturbation of the respective upstream regulator. Ranking and sorting the genes by decreasing scores, the analysis highlighted a small group of top-scoring, disease-associated regulatory genes, *Egr1*, *Klf6*, and *Klf4*. These genes and their associated proteins may serve as a basis for new putative drug target selections.

**Table 4:** Ranking of 8 key regulatory genes identified by the *in-silico* network perturbation analysis on the endothelial sub-network for male-specific DEGs. The scores assigned to each gene represent the total number of downstream genes in the Tg2576-associated regulatory network, whose gene expression can be reverted toward the WT expression pattern through the modulation of the corresponding upstream regulator (sorted from highest score on the left to lowest score on the right)

Gene	*Egr1*	*Klf6*	*Klf4*	*Hspb1*	*Nfkbia*	*Hes1*	*Edn1*	*Son*
Score	15	13	12	5	3	3	3	2

Specifically, the investigation revealed that the transcription factor *Egr1*, which apart from endothelial changes also displayed significant differential expression in microglia of both genders and significant gender-dimorphic AD-associated alterations in astrocytes (see above and Suppl. Table 2), attained the highest perturbation score. It has the potential to revert the gender-related expression changes of 15 genes in the Tg2576-associated regulatory network toward the gene expression profile of WT mice. Since other genes showed either a slightly or significantly lower potential to revert downstream gender- and disease-associated gene expression alterations (see Table 4), this analysis indicates that *Egr1* may provide the most promising candidate target in the affected regulatory sub-network for pharmacological modulation to alleviate Tg2576-associated male-specific pathological alterations, at least those linked to the most robust sub-network changes. However, the difficulty in targeting it in specific cell types might make this goal hard to achieve.

The transcription factor with the second highest perturbation score was *Klf6* (Kruppel-like factor 6), which, in addition to the altered expression in endothelial cells, also showed differential expression in microglial cells in both genders, and a male-specific decrease in neuroblast cells (see Table 2 and Supplementary Table 2). Its modulation can revert the expression of 13 downstream male-specific DEGs in the network. It has previously been implicated in the TGF-β signaling pathway, where it upregulates several of the pathway member genes [59], and in a survival pathway in hippocampal neurons, where its suppression promotes apoptosis [60]. Moreover, in retinal ganglion cells, over-expression of *Klf6* increased neurite outgrowth [61], suggesting a neurotrophic function for this gene. However, no prior reports on gender-specific differences in Klf6 expression could be found in the literature.

A further KLF gene, *Klf4*, reached the third highest perturbation score, and also corresponded to the transcription factor regulating the largest number of downstream genes in the GRN created in the combined analysis of male-specific DEGs across the different cell types (see Fig. 8 and the section on the regulatory network analyses). It controls 12 male-specific downstream DEGs and additionally displayed a significant male-specific decrease in microglial cells (see Supplementary Tab. 2). *Klf4* has been implicated in DNA damage response, mediating tumor suppressor p53-dependent G1-to-S cell cycle arrest [62]. In the brain, the protein Klf4 has been shown to protect microvascular endothelial cells from ischemic stroke through the activation of the gene *Malat1* [63]. In our study, while *Malat1* displayed a significant decrease in both genders in endothelial cells, the absolute log. fold change was larger in males (− 0.56) than females (− 0.34), and matched with the upstream decrease in the regulator *Klf4*, suggesting that the two factors are mechanistically linked also in Tg2576 mice. Another study showed that the protein Klf4 alleviates vascular injury after cerebral ischemic stroke by regulating the endothelial expression of inflammatory cell adhesion molecules [64], in line with the observed cell adhesion process alterations observed in our pathway enrichment analyses (see above and Supplementary Tab. S3). Finally, *Klf4* regulatory effects have also been linked to multiple neurophysiological and neuropathological processes in AD, including neuroinflammation, neuronal apoptosis, axon regeneration and iron accumulation, and the Klf4 protein was proposed as a drug target for neurodegenerative disorders [65].

Apart from the *Klf* family genes and *Egr1*, other transcription factors showed the ability to revert smaller subsets of downstream expression changes in the regulatory network, and have previously also been implicated in AD, including the chaperone-encoding *Hsbp1* [66–69], the NF-κB Inhibitor Alpha encoding gene *Nfkbia* [70–72], the transcription factor *Hes1* [73–75], and the vasoconstrictor endothelin-encoding *Edn1* [76–78] (see Table 4).

Overall, multiple regulatory genes with high scores in the differential expression and network analysis have previously been linked to AD. The top score for *Egr1* in the network perturbation analysis, the significant gender-dependent AD associations for this gene across multiple cell types, and its previous functional implications in AD derived from the literature, suggest *Egr1* as the main candidate target for further preclinical pharmacological research on reverting male-specific pathological endothelial changes in AD.

### Comparison with scRNA-seq Data for Human AD Patients and Controls

To compare the cell-type specific DEGs identified in the Tg2576 model of AD with transcriptomic changes in human AD, we investigated a single-cell RNA-seq dataset covering entorhinal cortex samples from AD patients and controls (6 per group), which has been described previously by Grubman et al. [10]. We applied the same analytical approach using a Poisson generalized linear model [17] as for the Tg2576 data to determine significant gender-neutral, gender-specific, and gender-dimorphic DEGs for each cell type, and then determined the overlap of significant DEGs across the mouse model and human dataset for the cell types present in both datasets (astrocytes, oligodendrocytes, endothelial cells, microglial cells, and neurons). Due to small cell counts observed for most cell types when categorized according to genders and conditions, a meaningful overlap analysis was only possible for astrocytes and oligodendrocytes. In astrocytes, we identified two gender-neutral DEGs, *NRXN1*, and *SLC1A2*, which displayed a shared significant under-expression after multiple hypothesis testing corrections in both genders and in both the Tg2576 model and human AD compared to controls (see Suppl. Table S4). In oligodendrocytes, one gene, *HSP90AB1*, showed a male-specific significant changes with different directionality in the Tg2576 model and in human AD (male-specific decreased expression in the Tg2676 model, and male-specific increased expression in human AD), and another gene, myelin basic protein (*M*BP), displayed a shared male-specific under-expression in both the Tg2576 model and the human AD data (see Suppl. Table S4 and the Discussion section).

## Discussion

In this study, we have uncovered gender-neutral, gender-specific, and gender-dimorphic DEGs, as well as gender-specific coordinated alterations in pathways and gene regulatory networks in one of the most commonly used transgenic mouse models of AD, the Tg2576 model. We found gender-dependent transcriptional changes that were cell-type specific and changes shared across all cell types combined, before the appearance of AD-like pathology, indicating that the disease follows at least in part differential trajectories depending on gender and cell identity.

### Gender-Neutral DEGs

Microglial cells displayed the largest number of significant gender-neutral DEGs, and 8 out of these 13 were transcription regulators (Fosb, Klf6, Atf3, Egr1, Jund, Mafb, Zfp36l1, and Chd4, see Suppl. Table 2). Microglia play a multitude of essential roles in the progression of AD pathology [79] and some mutations in microglial genes are known to increase AD risk [80]. A number of systems biology studies have highlighted the protective functions of microglia and proposed that a loss of microglial cell function may involve a loss of neuroprotection that could contribute to neurodegeneration [81, 82]. Our results indicate that microglial transcriptional regulation is significantly modified well before AD-like pathology can be detected in the brains of Tg2576 mice. This may have important implications for microglia-directed therapies [79].

Multiple significant gender-neutral DEGs were also detected in endothelial cells (11 DEGs in total, including the 6 transcriptional regulators *Fos*, *Fosb*, *Atf3*, *Jun*, *Jund*, and *Malat1*, see Suppl. Table 2). Endothelial cells are susceptible to Abeta and, in AD, are the site of vascular Abeta deposits [83]. The differential expression of mainly transcription regulators in these cells indicates that their transcriptional response to Abeta-induced stress is highly coordinated.

### Gender-Specific DEGs

Our study found more statistically significant expression alterations in Tg2576 males than in their female littermates. This in contrast to other studies, on mouse models or human samples, that found the opposite [9]. The standard deviation in the expression values across all genes and all cells and the average log. fold changes between transgenic and wildtype samples were slightly lower in females (stddev.: 0.11, average log. fold change: − 0.008) than in males (stddev.: 0.13, average log. fold change: − 0.014), suggesting that the lower number of detectable significant DEGs in females does not reflect higher variation, but smaller absolute log. fold-changes compared to males. It is important to note that we performed our study on mice that did not have AD-like pathologies yet. It is also important to note that, while women, at least at late age, have an increased risk for AD, men tend to have a more aggressive disease progression, and, curiously, mild cognitive impairment, a cognitive dysfunction that usually precedes AD diagnosis, is more pronounced in men (for review, see [5]). Finally, gene expression changes in susceptible brain regions may go through cycles that differ, temporally as well as qualitatively, between males and females as the disease starts and progresses. Most studies, including ours, provide a snapshot of gene expression profile at one particular time point.

### Gender-Dimorphic DEGs

One of the most interesting observations in our study was the presence of a small number of gender-dimorphic DEGs. These were genes whose transcription was significantly differentially expressed in Tg2576 mice compared to WT littermates in both genders, but in opposite direction in males versus females. *B2m* and *H2*-*D1* expression in endothelial cells, *Egr1* expression in astrocytes, and *mt*-*Rnr2* expression in neurons, were all significantly higher in Tg2576 females compared to their WT counterparts, whereas the opposite was true for males. Additionally, for *Egr1*, a gender dimorphism was also observed in the percentage of cells expressing the gene, which was increased in Tg2576 females compared to WT females, whereas the opposite was observed for males.

For the proteins encoded by all four gender-dimorphic DEGs, *B2m*, *H2-D1*, *mt*-*Rnr2* and *Egr1*, functional roles in an AD context have been reported (see following paragraphs). Our study highlights that their AD-dependent expression changes and, thus the roles of these factors, may be modulated by gender. Thus, since some of these proteins have been proposed as potential drug targets, possible future therapeutic interventions targeting a selection of these proteins or their associated cellular processes may have to be adjusted based on gender.

#### Gender-Dimorphic Gene 1–B2m

*B2m* encodes a component of MHC class 1 molecules, and has previously already been reported to undergo a CNS-wide sexually dimorphic induction, with a greater induction in females than males [41], in line with observed cell-type specific expression alterations in our study. The *B2m* gene codes for a negative regulator of the immune system with antimicrobial and phagocytic activities [84–86]. Several studies reported associations between *B2m*, AD, and natural aging: *B2m* protein levels are increased in the hippocampus of aged mice [87, 88], in the blood of AD patients compared to healthy controls, and non-AD mild cognitive impairment [37], and in the cerebrospinal fluid of AD patients compared to healthy controls [89]. Interestingly, injecting *B2m* systemically or in the hippocampus impairs cognitive function and neurogenesis in young mice, and genetically knocking down *B2m* in aged mice abrogates age-related cognitive decline and enhances neurogenesis [39]. Gender-associated differences in human B2M expression have not only been reported for the CNS [41], but also for serum of healthy individuals, where B2M levels were higher in males than females [90]. Thus, the Tg2576 mouse model may be well suited to study gender-related effects of *B2m* in an AD context.

#### Gender-Dimorphic Gene 2–H2-D1

*H2-D1* is an H-2 class I histocompatibility antigen, which directly interacts with *B2m* in the murine class I major histocompatibility complex H-2Db (see the crystal structure of the complex in the PDB database entry 5TJE). This matches with the observation that *H2-D1* undergoes the same type of gender dimorphic expression alterations as *B2m* in endothelial cells. While *H2-D1* has not been associated directly with AD, significant age-related changes for *H2-D1* have been reported [41, 91].

#### Gender-Dimorphic Gene 3–mt-Rnr2

The *mt*-*Rnr2* gene codes for a 16S mitochondrial subunit ribosomal RNA and for a micropeptide called humanin [92]. In the specific context of AD, the presence of humanin immunoreactivity has been confirmed in both normal and AD human brains, where it was found in large intact neurons of the occipital lobes and small and round reactive glial cells in the hippocampus [93]. Humanin has been shown to have multiple neuro- and cytoprotective functions, including increased survival and protection against oxidative stress in in vitro and in vivo models for AD, stroke, and Huntington’s disease [47–51], and suppression of apoptosis by binding to the protein Bax and blocking Bax translocation from the cytosol to mitochondria [45]. It was reported to reduce aggregation and fibril formation of the 42-aa form of Abeta (Abeta42) by suppressing the effect of Abeta42 on mononuclear phagocytes and competitively inhibiting the access of Formylpeptide-Receptor-Like 1 to Abeta42 [46]. Furthermore, independent studies showed that the plasma levels of humanin decrease significantly with age in both mice and humans, suggesting that it may play a role in aging-related processes [51, 94]. A close homolog of human *MT-RNR2*, the humanin-like protein 8 (*MTRNR2L8*), which differs from *MT-RNR2* only by a single residue and which has a polymorphism that causes it to be identical to *MT-RNR2* [95], was previously found to display significantly increased expression in excitatory neurons with AD pathology vs. no pathology in prefrontal cortex samples from human subjects (*q* value = 2.9E-182; this result derived from single-nucleus RNA-seq data was also confirmed by qPCR by the authors) [9]. In the same dataset, AD-associated neuronal cell subpopulations were enriched with female cells, including the sub-population of excitatory neurons for which *MTRNR2L8* was identified as a marker gene. The gender-dependent expression observed here for murine *mt-Rnr2* is also line with a previously reported inhibitory action of estradiol on humanin expression in pituitary cells [96]. Thus, *MT-RNR2* may be of interest for further research in the context of sex-specific differences in AD, and additional studies in independent human biospecimens and further AD model systems, such as the 3xTg mouse model, are warranted to corroborate the previous findings and further elucidate the potential mechanistic role of *MT-RNR2* in AD.

#### Gender-Dimorphic Gene 4–Egr1

The only gene with a significant gender-dimorphic change in astrocytes was *Egr1*. *Egr1* was also globally differentially expressed, with a shared decrease in Tg2576 mice in both genders, a pattern likely driven by its sharply lower expression in microglia. Moreover, *Egr1* showed a male-specific decrease in endothelial cells. Finally, in the endothelial gene regulatory network perturbation analysis, *Egr1* was determined as the top-scoring transcriptional regulator, whose modulation could maximally shift the transcriptional profile of Tg2576 mice toward that of their WT littermates. Multiple lines of evidence indicate that *Egr1*/*EGR1* is worth investigating as a drug target for AD. In the 3xTg-AD mouse model, silencing *Egr1* in the hippocampus by shRNA has been reported to reduce tau phosphorylation, lower Abeta pathology, and improve cognition [44]. In the same model, as well as in human AD, mRNAs for *Egr1/EGR1* and for the enzyme acetylcholine-esterase (AchE) correlate, and in vitro, EGR1 upregulates AchE [97]. Since EGR1 is elevated in early AD, it could contribute to the loss of acetylcholine typical for this disease [97]. EGR1 upregulates presenilin-2 in neuronal cells, thus possibly promoting the amyloidogenic processing of APP [98]. A study using mouse brain in situ hybridization (ISH) data from the hippocampus to reverse-engineer AD-specific transcriptional regulatory networks identified *Egr1* as a key regulator of genes known to be involved in AD pathogenesis [99]. A further independent study applied ChIP-seq analysis to discover *Egr1* target genes in the brain of an AD mouse model (APP23), and identified and validated genes directly implicated in AD as Egr1 targets (such as *Picalm*, *Psen2*, and *App*), as well as genes associated with synaptic plasticity and protein transport (such as *Arc*, *Grin1*, *Syn2*, *Vamp2*, and *Stx6*) [100]. The same study also demonstrated an association in vivo related to the gene’s role in memory formation, showing that a spatial memory test which leads to an up-regulation of *Egr1* levels in the brain, also results in increased expression of its target genes. As for most targets, an optimal level of inhibition would have to be determined, since prior evidence suggests excessive inhibition is detrimental. Specifically, the MGI mouse knockout database reports that homozygote mice for targeted mutations in *Egr1* display memory deficits (www.informatics.jax.org/marker/MGI:95295).

Human genetic studies on *EGR1* (public database GTEx, Analysis Release V8) report a significant eQTL for human *EGR1* (dbSNP ID: rs115259732), which is associated with altered gene expression of *EGR1* in the cortex, but this variant has not yet been investigated in the context of AD. However, a truncating mutation in the *EGR1* gene has previously been proposed to play a pathogenic role in intellectual disability (c.1347_1348insA) [101]. Epigenetic studies of *EGR1* have also revealed roles in brain function and development. It is regulated epigenetically by the amyloid precursor protein (APP), both in vitro and in vivo, and its transcription is increased following exposure of APP + / + mice to novelty, while a high basal level of *Egr1* expression in in APP − / − mice prevented this induction [102]. Whether this finding has implications for AD is unclear. In the context of other brain disorders, *Egr1* has been shown to regulate the expression of the glial scar component phosphacan in astrocytes after experimental stroke [103], and to push neurodegeneration and neuroinflammation in a model for Parkinson’s disease [104].

A couple of studies have pointed out gender-specific effects of Egr1 in the brain. Egr1 was shown to be involved in mediating influences of the estrous cycle on the adult rat medial prefrontal cortex, and to bind to synapse-related genes in a gender-specific and estrus cycle-specific manner [105]. Moreover, *Egr1* expression in the rat prefrontal cortex has been implicated in controlling gender differences in social anxiety behaviors [106].

Taken together, all these findings indicate that inhibition of *Egr1* expression or of its protein action may be worth further investigation as an adjuvant treatment strategy for AD, with a greater potential in females than males due to the gender-dependent changes across multiple cell types. Our data furthermore indicate that in females, a cell-specific inhibition in astrocytes may be of particular interest for further study. Although this is feasible to implement pre-clinically by a cell type-specific knockdown in mice, it may be very hard to achieve in the clinic. A small molecule compound, named ML264, that potently inhibits the expression of *Egr1* has previously already been identified [107]. A brain-penetrant formulation of that compound could serve as a tool for preclinical follow-up studies on *Egr1* activity modulation in AD models.

### Network Analyses

According to the network perturbation analyses, a further possible target gene that could shift the downstream expression patterns in the transcriptional profile of Tg2576 toward those of WT littermates is the transcription factor *Klf6*. The gene itself has emerged as a gender-neutral DEG in the global analysis across cell types and in microglial cells. It also showed a significant male-specific decrease in endothelial cells and neuroblast cells, contributing to the regulation of genes with male-specific expression changes in endothelial cells according to the regulatory network.

The major cellular source of *Klf6* in mouse brains at baseline are astrocytes and endothelial cells (https://www.brainrnaseq.org). In human brains, *KLF6* is mostly expressed in the midbrain and spinal cord at tissue level, and in neuronal cells at cellular level (https://www.proteinatlas.org). Klf6 has potent proinflammatory properties in cells of the myeloid lineage [108, 109]. Its decreased expression in microglia may therefore be a protective response to Abeta, and further inhibition may help mitigate an excessive microglial reactivity that contributes to neurodegeneration. However, here too, a cell-specific inhibition may be the best, yet most challenging, approach to investigate, since neuronal Klf6 has been reported in several studies to have neurotrophic and neuronal survival properties [60, 61, 110]. Interestingly, a regulatory variant for human *KLF6* (dbSNP ID: rs10795076), located in a transcription factor binding site known as the CArG box, causes decreased promotor activity [111]. Multiple eQTLs for *KLF6* also alter its expression in the cortex (GTEx database, Analysis Release V8), but these variants have not yet been investigated in the context of AD.

While mouse models for chronic, age-related neurodegenerative disease open up a window into hitherto unseen features of a disease, such as its earliest phases, they have their limitations. For instance, humans, but not rodents, experience, as they age, a dramatic decrease in circulating gonadal hormones (for review, see [112]). It is unclear how this affects AD initiation and progression, since, thus far, no effect of hormonal replacement therapy on AD could be demonstrated [5].

Transgenic mouse AD models typically overexpress just one or at best a couple of familial AD-linked genes and are mostly just on one (usually Bl6) or dual hybrid (such as in this study, Bl6 and SJL) genetic background. Future studies will have to extract cell-type specific core genetic signatures of pre-AD-like disease, by comparing the one we describe here with that of other AD models (such as tau or ApoE3/E4 based), and parse them out based on gender. It may also be necessary to compare a multitude of strain backgrounds to more truly reflect the variations observed in humans [113]. Such studies, while they are quite resource-intensive and time-consuming, are currently ongoing in our and other labs.

Overall, across different bioinformatics analyses of single-cell RNA-seq data, we have identified significant gender-associated expression alterations in the Tg2576 mouse model of AD compared to WT controls, both at the level of individual genes and at the level of cellular pathway and regulatory network changes. The results reveal statistically significant gender-dimorphic changes in genes previously linked to AD and aging, including *B2m* and *Egr1*, and an enrichment of gender-related changes in nervous system related cellular pathways associated with synapse organization, reactive oxygen species metabolism, and neurotransmitter metabolism. A regulatory network analysis for endothelial cells in particular also highlighted the roles of the transcription factors *Egr1* and *Klf6* in controlling many of the observed downstream genes displaying male-specific transgene-associated alterations. Finally, a network perturbation analysis suggested that the pharmacological modulation of *Egr1* may have the potential to revert many disease- and gender-associated transcriptomic changes in the Alzheimer model toward the wildtype control phenotype, in line with previous reports on protective effects of *Egr1* inhibition in other AD model systems. While an scRNA-seq dataset for human AD patients and controls provided too small cell counts per condition, gender and cell types for a comprehensive comparison with the Tg2576 data, in a first analysis of overlapping DEGs we identified two shared gender-neutral DEGs in astrocytes, *NRXN1* and *SLC1A2*, and two shared male-specific DEGs in oligodendrocytes (one with consistent decreased expression, *MBP*, and one with opposite directionality in the Tg2576 model and in human AD, *HSP90AB1*, see Suppl. Table S4). Myelin basic protein (*MBP*) encodes a main constituent of the myelin sheath of oligodendrocytes in the nervous system, which has been reported to associate with AβPP, Aβ1-42, and amyloid plaques in the cortex of AD brains in response to AD-linked axonal and myelin injury [114]. Hormone-mediated sex differences in *MBP* levels have previously already been observed in the orbital frontal cortex in rats, were males showed significantly lower *MBP* levels than females, consistent with our findings [115, 116]. The second gene with male-specific alterations in oligodendrocytes, *HSP90AB1* (also known as *HSP90beta*) codes for a chaperone belonging to the heat shock protein 90 family, which has been implicated in AD due to its role in the stabilization of the AD-associated tau protein [117]. Among the gender-neutral DEGs in astrocytes, *NRXN1* (Neurexin 1) encodes a receptor that binds neuroligins to form Ca(2 +)-dependent neurexin/neuroligin complexes at synapses in the central nervous system, a process affected by synaptic damage in AD [118]. Finally, *SLC1A2* (also known as *EAAT2* and *GLT1*), the second gender-neutral DEG in astrocytes, codes for a transporter responsible for glutamate clearance from the extracellular space at synapses. Dysregulation of *SLC1A2* and other glutamate transporters has been implicated in multiple neurodegenerative disorders, including AD, where its astrocytic deficiency has already been reported previously [119, 120]. For other cell types, the cell counts in the human scRNA-seq dataset were significantly lower, and follow-up studies with larger sample sizes will be required to assess whether further shared cell-type specific DEGs between the Tg2576 model and human AD can be identified. The mouse and human datasets also differed in terms of the disease stages they represent, with the Tg2576 model reflecting early AD-like characteristics and the human data representing late-stage AD. Thus, future investigations on human data will not only require larger sample sizes, but should also cover samples from earlier stages of AD.

It is important to stress that the multitude of molecular changes observed in our animal study occur before AD-like pathologies can be detected in the Tg2576 mice. These observations lay the ground for future follow-up studies on the involvement of *Egr1*, *B2m*, and *Klf6* gene products in AD pathogenesis and progression, and exploring the effects of pharmacological inhibition and activation of these target proteins on AD pathology.

## Materials and Methods

### Animals

Twenty-four-week-old Tg2576 mice (B6;SJL-Tg(APPSWE)2576Kha) heterozygous transgenic mice of both genders, and wildtype littermate controls, were purchased from Taconics (9 per genotype/gender). Details can be found on the Taconic website (https://www.taconic.com/transgenic-mouse-model/appswe-model-1349). Mice were housed in dedicated facilities under specific pathogen-free conditions for 1 week to acclimatize. Mice always had ad libitum access to standard mouse food (Ssniff, # V 1534–300) and water. All mouse experiments were performed according the European FELASA guidelines for animal experimentation and had been approved by the local institutional Animal Experimentation Ethics Committee, and by the overseeing Luxembourg government agencies (Ministry of Agriculture, Ministry of Health). After deep anesthesia (150 mg kg ketamine, 1 mg kg metedomidine, i.p.), mice were transcardially perfused with PBS to remove the blood, then brain were quickly removed from the skull. Euthanization took place in the middle of the day (between 10 am and 3 pm) in a random order, i.e., no particular group was euthanised before the other in order to exclude any potential systematic difference related to circadian rhythm. The reader should however note that circadian effects can still lead to global shifts in gene expression, i.e., affecting all collected samples in the same manner, and that in order to obtain analysis results that are representative also for other time periods in the circadian cycle (e.g., at night time) further longitudinal follow-up studies would be needed. One hemibrain was fixed for histology (see below), and the other was dissected into brain regions. Dissected cortex was used for single cell DropSeq (see below). Mice genotypes were confirmed at the protein level after euthanasia by immunohistochemistry for the transgene (human Amyloid Precursor Protein, hAPP, not shown).

### Single Cell Sequencing

#### Tissue Extraction and Cells Isolation

Mice were euthanized in deep anesthesia with the administration of medetomidine (1 mg/kg) and ketamine (150 mg/kg) and transcardially perfused with PBS. The mouse cortex was dissected manually, following by its dissociation using the Adult–Brain Dissociation Kit (Miltenyi Biotec) according to manufacturer’s instructions. Cells were then resuspended in 0.5% BSA and Drop-seq analysis was applied to obtain single cell RNA-seq (scRNA-seq) data (see following sections).

### Drop-seq Analysis and Library Preparation

#### Microfluidics Devices

Microfluidics devices were generated using a previously published design [121]. Soft lithography was performed using SU-8 2050 photoresist (MicroChem) on 4′′ silicon substrate to obtain a feature aspect depth of 90 μm. After overnight silanization (using chlorotrimethylsilane; Sigma), the wafer masks were used for microfluidics fabrication. Drop-seq chips were generated using silicon-based polymerization chemistry, according to a previously published protocol [122]. Briefly, polydimethylsiloxane (PDMS) base and cross-linker (Dow Corning) were mixed at a 10:1 ratio, and degassed before pouring the solution onto the Drop-seq master template. PDMS was cured on the master template, at 70 °C for 2 h. After incubation and cooling, PDMS monoliths were cut and the inlet/outlet ports were punched with 1.25-mm biopsy punchers (World Precision Instruments). The PDMS monolith was plasma-bonded to a clean microscopic glass slide using a Harrick plasma cleaner. After pairing the plasma-treated surfaces of the PDMS monolith and the glass slide, flow channels of the Drop-seq chip were subjected to a hydrophobicity treatment using 1H,1H,2H,2H-perfluorodecyltri-chlorosilane (in 2% v/v in FC-40 oil; Alfa Aesar/Sigma). After 5 min of treatment, excessive silane was blown through the inlet/outlet ports. Chips were further incubated at 80 °C for 15 min.

#### Single-Cell Droplet Encapsulation

Single cell RNA sequencing was performed following the original Drop-seq protocol [121] as previously described [123], with the following modifications detailed below. Synthesized barcoded beads (ChemGenes Corp., USA) were co-encapsulated with cells inside the droplets containing lysis reagents using an optimal bead concentration of 180 beads/μl in Drop-seq lysis buffer medium. Cellular mRNA was captured on the beads via barcoded oligo (dT) handles synthesized on the surface.

Dissociated cells were filtered with 40 μm strainer, counted and assessed to confirm their viability > 80%, and corrected for cell concentration of 120 cells/μl. For cell encapsulation, 1.5 ml of bead suspensions and cell suspension were loaded into 3-ml syringes (BD). To keep beads in homogenous suspension, a micro-stirrer was used (VP Scientific). The QX200 carrier oil (Bio-Rad) used as continuous phase in the droplet generation was loaded into a 20-ml syringe (BD). For droplet generation, 2.5 ml/h and 11 ml/h flowrates were used in KD Scientific Legato Syringe Pumps for the dispersed and continuous phase flows, respectively. After stabilization of droplet formation, the droplet suspension was collected into a 50-ml Falcon tube. Collection of the emulsion was carried out until 1 ml of the single-cell suspension was dispensed. Droplet consistency and stability were evaluated by bright-field microscopy using INCYTO C-Chip Disposable Hemacytometer (Thermo Fisher Scientific). Bead occupancy within the droplets was carefully monitored to avoid multiple beads per droplet.

The subsequent steps of droplet breakage, bead harvesting, reverse transcription, and exonuclease treatment were carried out in accordance with the Drop-seq method [121]. RT buffer contained 1 × Maxima RT buffer, 4% Ficoll PM-400 (Sigma), 1 μM dNTPs (Thermo Fisher Scientific), 1 U/ml RNase Inhibitor (Lucigen), 2.5 μM Template Switch Oligo [121], and 10 U/ml Maxima H-RT (Thermo Fisher Scientific). After Exo-I treatment, the bead counts were estimated using INCYTO C-Chip Disposable Hemacytometer, and 10,000 beads were aliquoted in 0.2 ml Eppendorf PCR tubes. PCR mix was dispensed in a volume of 50 μl using 1 × HiFi HotStart ReadyMix (Kapa Biosystems) and 0.8 mM Template Switch PCR primer. The thermocycling programme for the PCR amplification was modified for the final PCR cycles to 95 °C (3 min), 4 cycles of 98 °C (20 s), 65 °C (45 s), 72 °C (3 min), and 9 cycles of 98 °C (20 s), 67 °C (20 s), 72 °C (3 min), followed by a final extension step of 72 °C for 5 min. After PCR amplification, libraries were purified with 0.6 × Agencourt AMPure XP beads (Beckman Coulter), according to the manufacturer’s protocol. Finally, the purified libraries were eluted in 10 μl RNase/DNase-free Molecular Grade Water. Quality and concentration of the sequencing libraries were assessed using Bioanalyzer High Sensitivity Chip (Agilent Technologies).

#### NGS Preparation for Drop-seq Libraries

The 3′ end enriched cDNA libraries were prepared by tagmentation reaction of 600 pg cDNA library using the standard Nextera XT tagmentation kit (Illumina). Reactions were performed according to the manufacturer’s instructions. The PCR amplification cycling programme used was 95 °C 30 s, and 12 cycles of 95 °C (10 s), 55 °C (30 s), and 72 °C (30 s), followed by a final extension step of 72 °C (5 min). Libraries were purified twice to reduce primers and short DNA fragments with 0.6 × and 1 × Agencourt AMPure XP beads (Beckman Coulter), respectively, in accordance with the manufacturer’s protocol. Finally, purified libraries were eluted in 10 μl Molecular Grade Water. Quality and quantity of the tagmented cDNA library were evaluated using Bioanalyzer High Sensitivity DNA Chip. The average size of the tagmented libraries prior to sequencing was between 400 and 700 bps.

Purified Drop-seq cDNA libraries were sequenced using an Illumina NextSeq 500 machine with the recommended sequencing protocol, except for 6 pM of custom primer (GCCTGTCCGCGGAAGCAGTGGTATCAACG CAGAGTAC) applied for priming of read 1. Paired-end sequencing was performed for the read 1 of 20 bases (covering the random cell barcode 1–12 bases and the rest 13–20 bases of random unique molecular identifier (UMI) and for read 2 of 60 bases of the genes. Raw reads were further demultiplexed and processed using a dedicated Drop-seq computational pipeline [121].

## Computational Analyses

### Data Pre-Processing and Quality Control

The single-cell transcriptomic data measured on the Illumina NextSeq 500 machine was pre-processed and analyzed following a standard workflow [17] and using the Seurat software package (version 3.1.4) in R (3.6.1). All samples passed the quality filters and were retained for the subsequent data analysis steps; the processed data covers 3337 single cells in total. Briefly, the data processing pipeline involves the selection and filtration of cells according to commonly used quality control metrics (see filters described below), the data normalization and scaling, the filtering of low-variation genes, and the statistical analysis of differentially expressed genes between transgenic and wildtype mice for both genders separately and combined. Outlier cells with unique feature counts over 2500 or less than 200 were filtered out to avoid potential problems of cell-doubling or empty droplets being sequenced. Moreover, we filtered out cells with more than 5% mitochondrial gene counts, since high expression levels of mitochondrial genes can be an indicator of apoptotic or lysing cells. Finally, for data normalization we employed a global-scaling normalization method that adjusts the feature expression measurements for each cell by applying a centered log ratio (CLR) transformation.

### Data Integration, Filtering, and Scaling

As this study involved two genotypes, wildtype (WT) and transgenic (TG), and considers gender-specific changes, in addition to cell-types specific analyses, we also performed an integrated analysis on all cell types to identify shared alterations across cell types and increase the statistical power for comparative analyses. For this purpose, we first identified anchors using the *FindIntegrationAnchors* function in the Seurat software package, and then used the pre-computed anchors to integrate the data with the *IntegrateData* function.

Next, in order to filter out low-variance genes with limited information content, we ranked all genes by cell-to-cell variation using the *FindVariableFeatures* function. In line with recommendations from the authors of the Seurat software, we retained only the top 2500 most variable genes for further downstream analyses. As a further standard pre-processing step, we used the *ScaleData* function with default parameters to apply a linear scaling transformation.

### Dimension Reduction, Clustering, and Cell-Type Annotation of the Identified Clusters

A dimension reduction was applied to the pre-processed data using a principal component analysis (PCA). The so-called elbow method, as implemented in the *ElbowPlot* function in the software package Seurat [17], was used as a heuristic to determine the optimal number of principal components (PCs) to extract for further analysis (in this case, 10 PCs). Then, a shared nearest neighbor (SNN) graph-based clustering [19] was conducted to assign the cells to different groups according the similarity of their expression profiles. Briefly, this approach embeds all data points into a graph structure, where edges connect cells with similar expression patterns, and then the graph is partitioned into strongly interconnected subnetworks representing clusters of cells that tend to reflect different cell types. The number of clusters was determined by scoring the clustering results for different possible cluster number settings using an internal validity index, the Silhouette Width, which accounts for both the separation between clusters and their compactness [20]. Finally, the functions *FindNeighbors* and *FindClusters* from the Seurat package (version 3.1.4) [17] were applied to identify clusters of cells, which were subsequently annotated for different cell types.

To obtain unique cell type annotations for each cluster, cell-type specific marker genes with pronounced expression in each cluster were determined using the Cell Marker database [22]. Using these cell-type specific marker genes, all identified clusters were annotated with a unique cell type.

### Identification of Genes with Gender-Specific and Gender-Dimorphic Differences Between Genotypes

In order to determine and rank the differentially expressed genes (DEGs) between conditions for both individual cell-type clusters and across different cell types, we used a Poisson generalized linear model approach [17], as implemented in the *FindMarkers* function from the Seurat software package. First, gender-specific differentially expressed genes (DEGs) between genotypes were identified, i.e., genes which are significant in only one gender (*q* value < 0.05), and not approaching significance in the other gender (*q* value > 0.5). For this purpose, transgenic (TG) samples for each gender were compared against their respective wild type (WT) samples from the same gender, considering each cell type and gender separately. The intersection of the two sets of DEGs obtained for each gender was computed for each cell type cluster, providing us both with the final lists of gender-shared DEGs (*q* value < 0.05 in both genders, same direction of the change), gender-dimorphic genes (*q* value < 0.05 in both genders, opposite direction of the change), and non-overlapping, gender-specific DEGs (*q* value < 0.05 for one gender, and *q* value > 0.5 for the other gender) for each cell type.

### Pathway Analyses

Next, in order to identify and characterize the cellular pathways and processes with altered activity in response to AD-associated molecular gender differences, we used the clusterProfiler R package (3.12.0) [124] to perform gene set enrichment analyses for the KEGG and Gene Ontology databases. All pathways with an adjusted *p* value (*q* value) < 0.05 were considered as significant, and the significant pathways identified were further characterized in terms of the number of mapped DEGs in relation to the number of pathway members, and the gene ratio, i.e., the size of the overlap of the gender-related DEGs with the pathway gene set in relation to the size of the overlap with all the members of the collection of pathway-representing gene set.

### Regulatory Network Construction

For the network analyses, we used the MetaCore (Clarivate Analytics) database to retrieve known, directed functional interactions between the genes displaying significant AD-associated molecular gender differences. MetaCore contains literature-curated and experimentally validated gene–gene interactions, with directionality information for regulatory interactions, enabling the construction of robust evidence-based gene regulatory networks. To obtain an appropriate set of functional regulatory interactions between the identified DEGs, our analysis was restricted to interactions belonging to the categories “Influence on Expression,” “Transcriptional Regulation,” “Regulation,” “co Regulation of Transcription,” and “Binding.” All extracted interactions are directed, i.e., the source and target genes are always known. Furthermore, when information about the interaction type (activation or inhibition) was available, it was considered in the network construction and used to annotate the interactions.

For the reconstruction of phenotype-specific networks for disease (TG) and control (WT) phenotypes, we employed an in-house developed differential GRN reconstruction approach [54]. Briefly, this approach relies on a genetic algorithm to remove interactions that are not compatible with Booleanized gene expression states of the disease and control phenotypes. As some of the interactions retrieved from MetaCore have an unspecified effect, i.e., information on the activating or inhibitory consequence of the interaction is missing, the software infers missing regulatory effect data from the given gene expression data and network phenotype under consideration.

### Network Perturbation Analyses

For the in-silico network perturbation analyses, we first identified the network perturbation candidate genes using the dedicated algorithm by Zickenrott et al. [54]. With the same software, we then performed a network simulation analysis by perturbing unique candidate genes and analyzing the downstream network effects. As a result, a ranked list of perturbed genes and their corresponding perturbation scores was obtained, which represent the number of downstream genes in the network whose expression change in the studied condition is predicted to be reverted through the chosen perturbation. Generally, a high score is indicative of a gene’s ability to regulate and revert the expression of a large subset of downstream genes; hence, playing a key role in the maintenance and stability of the phenotype under consideration.

### Immunofluorescence Analyses

One hemibrain of each mouse was used for immunohistochemistry. The genotype was confirmed by checking the human APP transgene (see below). Extracted hemibrains were fixed in in 4% buffered PFA for 48 h and kept in PBS with 0.02% NaN_3_ until they were cut with a vibratome (VT1000 S from Leica) into sagittal 50 µm free-floating sections. Before the staining procedure, sections were kept at – 20 °C in a cryoprotectant medium (1:1 v/v PBS/Ethylene Glycol, 10 g L^−1^ Polyvinyl Pyrrolidone).

All staining steps were performed at room temperature. Sections were washed 3 × in PBS between each incubation step. To block endogenous peroxidases and for permeabilization, sections were incubated with 3% H_2_O_2_ v/v and 1.5% Triton _X100_ v/v for 30 min. To avoid unspecific antibody binding, sections were incubated with 5% BSA w/v in PBS for 1 h before they were incubated with the primary antibody overnight in 2% BSA (anti-hAPP (Sigma, 1/1000, anti-hAPP (Invitrogen 1:1000)—both giving similar results); anti total APP clone 22C11 (Thermofisher, 1:1000), anti Abeta clone 82E1 (IBL, 1/500), anti IBA1 (FUJIFILM WAKO, 1/1000). The following day, sections were incubated with an appropriate secondary fluorophore-coupled antibody (donkey anti-rabbit alexa 647 for visualization of hAPP, donkey anti-mouse alexa 488 for visualization of Abeta plaques, and goat anti-rabbit alexa 647 for visualization of microglia), all diluted 1/1000. Fluorescently stained sections were mounted on Superfrost plus slides (Thermoscientific, Walham, MA), air-dried, and coverslipped using ProLong Gold antifade mountant (Life technologies, Darmstadt, Germany). Fluorescence signals were acquired using a Zeiss Axio Imager Z1 at 20 × magnification.

To quantify total and hAPP levels in the cortex, for each marker, 2 random sections per mouse, and 3 optical fields (227 × 163 µm) per section, were imaged with an epifluorescence microscope (Zeiss AxioImager Z1 equipped with Zen Blue software) at 40 × magnification. For total APP quantification, because the immunofluorescent signal was very weak in WT mice, we collected a Z projection of 3 pictures at each optical field and used it to generate one intensity measurement. The mean grey intensity of each resulting picture was determined for each Z-stack using the FIJI software. For quantification of hAPP in the cortex, we analysed a single plane picture for each optical field.

To quantify microgliosis in the cortex, we used 2 sections/mouse, and acquired 3 cortical pictures (image dimension: 447 × 335 μm) for each section. Using the open source Fiji software, the percentage area occupied by the immunoreactive signal was measured in the defined ROI with the thresholding algorithm by Huang et al. [125]. Values for the 6 images obtained for each mouse were averaged.

For APP, hAPP, and Iba1 staining and quantifications, all image acquisitions and measurements were done with blind-coded sections, and codes were not broken until the analysis was complete.

Statistical analysis was done with PRISM (Graphpad, San Diego, CA), using two-way ANOVA (gender genotype). Post-hoc analysis, where appropriate, was done using the Tukey post-hoc test.

### Supplementary Information

Below is the link to the electronic supplementary material.

Supplementary file1 (PDF 4918 KB)

Supplementary file2 (XLSX 130 KB)

Supplementary file3 (XLSX 32 KB)

Supplementary file4 (XLSX 98 KB)

Supplementary file5 (XLSX 12 KB)

## Data Availability

Supporting data for the manuscript has been included as electronic supplementary material.
