# Supplementary material for: Single-Cell Transcriptional Profiling and Gene Regulatory Network Modeling in Tg2576 Mice Reveal Gender-Dependent Molecular Features Preceding Alzheimer-Like Pathologies
Source: Mol Neurobiol. 2022 Aug 18;61(2):541–66. doi: 10.1007/s12035-022-02985-2 (PMC10861719; doi:10.1007/s12035-022-02985-2)
Supplement: Supplementary file 1 — Supplementary file1 (PDF 4918 KB) [file 12035_2022_2985_MOESM1_ESM.pdf]

Supplementary Figures

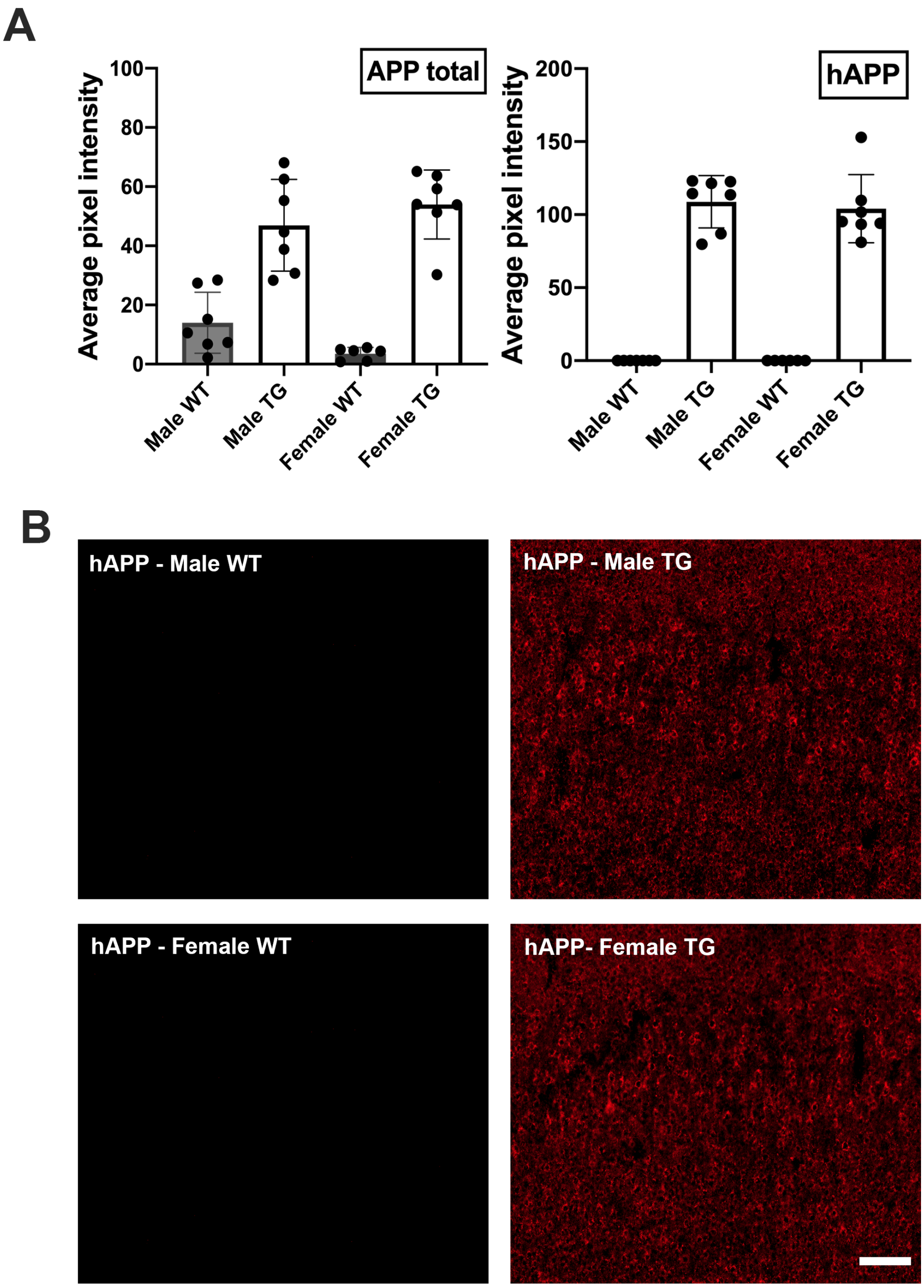

Fig. S1: A. Intensity quantification of immunofluorescent signals of total APP and hAPP in WT and TG (Tg2576) mice used in this study. No significant gender differences were found for total APP (the apparent differences in WT mice are not statistically significant). Similarly, no significant differences were found for hAPP for TG mice (no signal above background was detected in WT mice). Bars show means +/- S.D. Statistical comparisons were done using ANOVA followed by Tukey's post-hoc test. For method details, see the main text. **B.** Representative microphotographs of hAPP staining in the cortex of male and female WT and TG mice. The stainings were similar to those reported in another

hAPP transgenic model for AD (PMID: 25592972). Note the staining around cells with neuronal morphology. Scale bar: 80 μm.

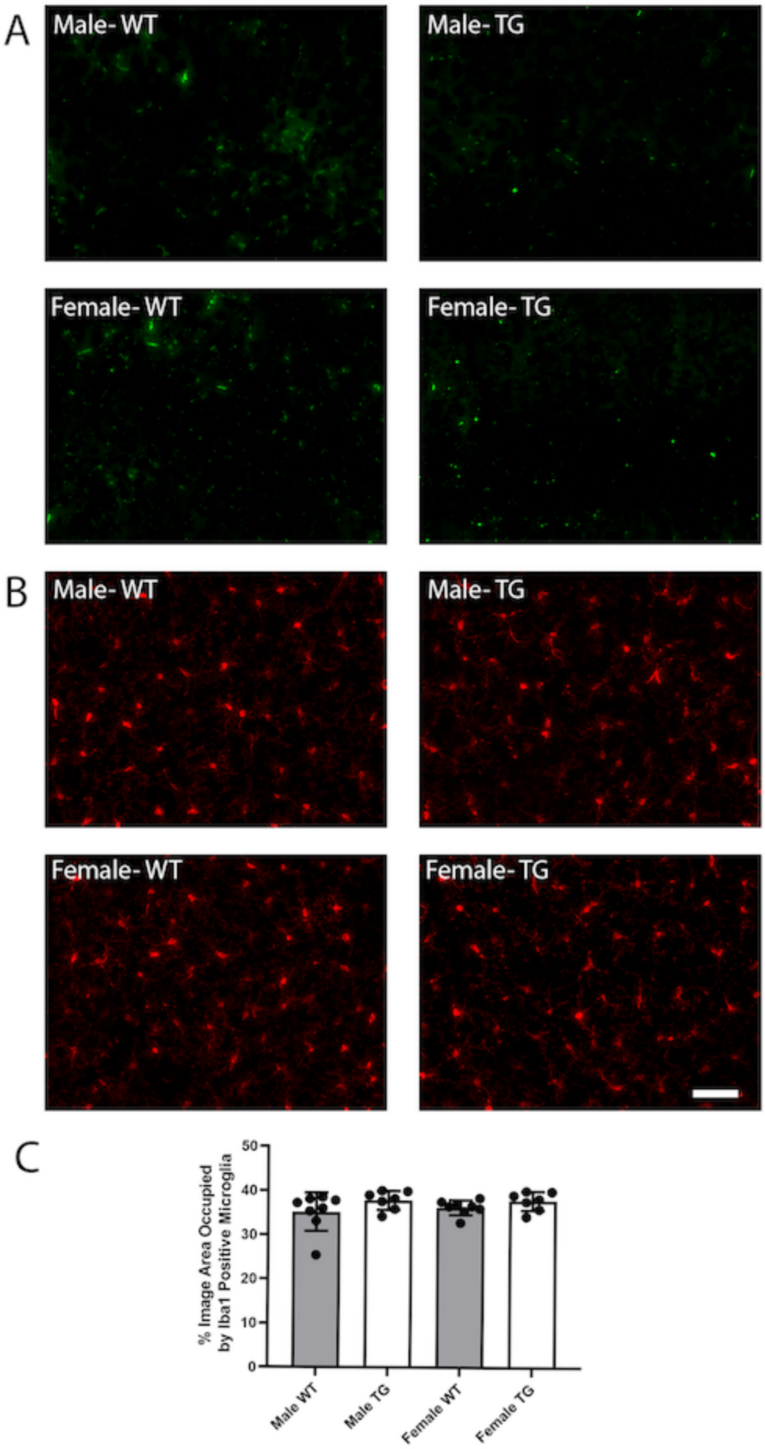

Fig. S2: Absence of Abeta plaques and microgliosis in the cortex of 24 weeks-old Tg2576 mice. Panels in A show immunofluorescent staining for Abeta, and panels in B that for the microglial marker Iba1 (see Materials and Methods for details), in male and female heterozygous Tg2576 mice (TG) and wildtype littermates (WT). The green dots and rods in the panels in A were found in both TG and WT mice are due to autofluorescence in the green channel and/or to non-specific binding of the secondary antibody to blood vessels. Scale bar = 60 micrometers. Panels C shows the

quantification for Iba1-positive microglia in the cortex of Tg2576 mice and wildtype controls (see Materials and Methods for details). No uptick in Iba1 signal was detected in Tg2576 mice at this age. Similar results (absence of plaques and microgliosis) were obtained for the hippocampus (not shown).

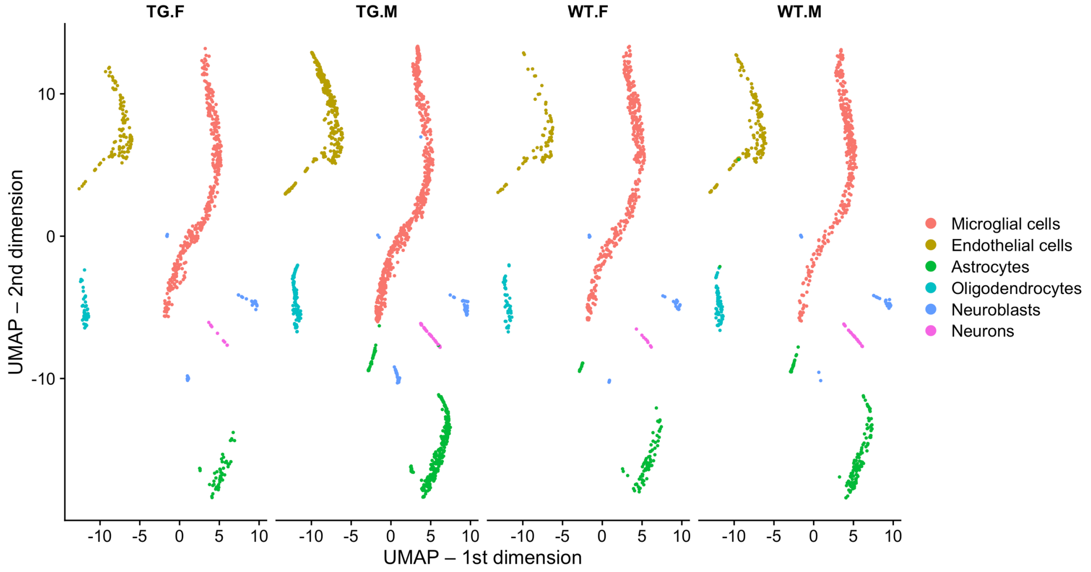

Fig. S3: Two-dimensional cluster visualization of the single-cell RNA-seq data with added condition annotations for all mice across both genders, male (M) and female (F), and genotypes, transgenic (TG) and wild type (WT).

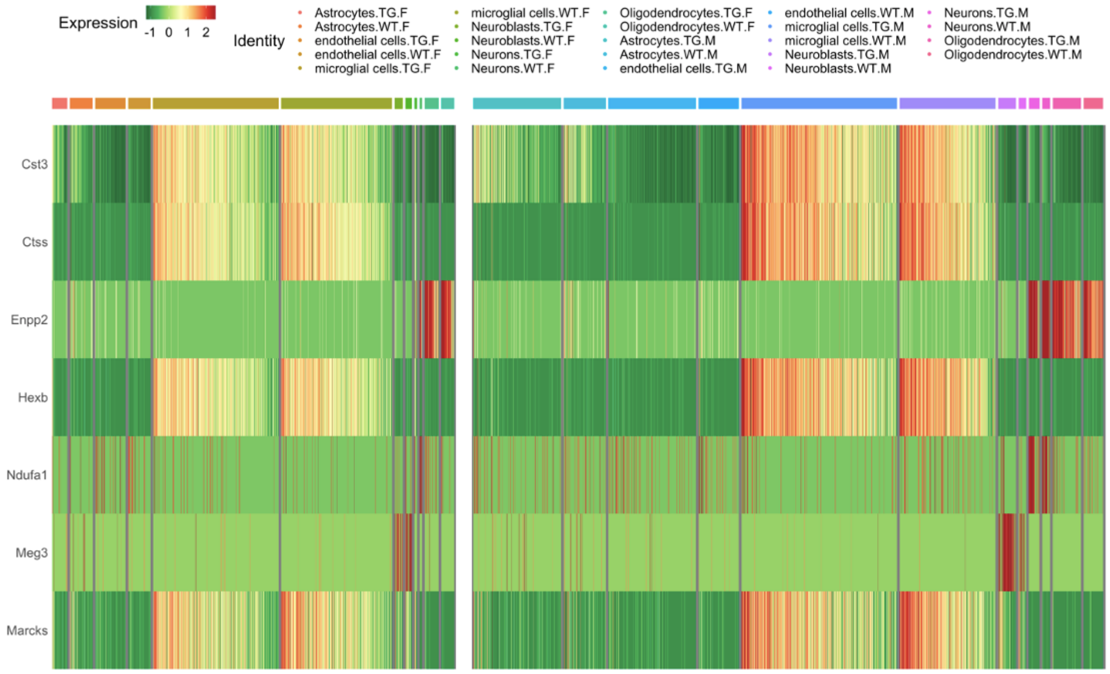

Fig. S4: Heat map visualization of the gene expression for the top 7 DEGs in terms of the absolute log fold-change across all cell types. The heatmap for the expression changes in female (left side) and male (right side) samples is

separated by a white partition in the middle. The colors at the top represent the cell type, the colors in the heat map represent the expression (see legends on the right).

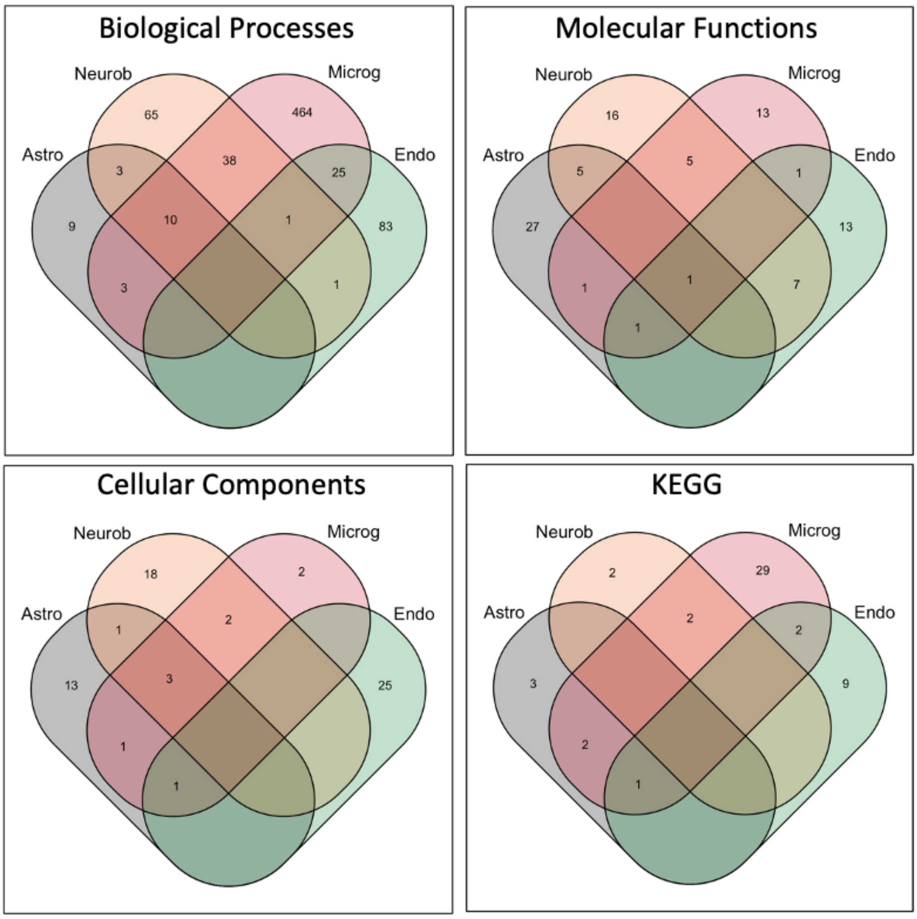

Fig. S5: A Venn diagram showing the number of unique and overlapping cellular processes with gender-specific or gender-dimorphic differences in AD that are common to all cell types as well as specific to one particular cell type (Microg = microglial cells, Endo = endothelial cells, Astro = astrocytes, and Neurob = neuroblasts).

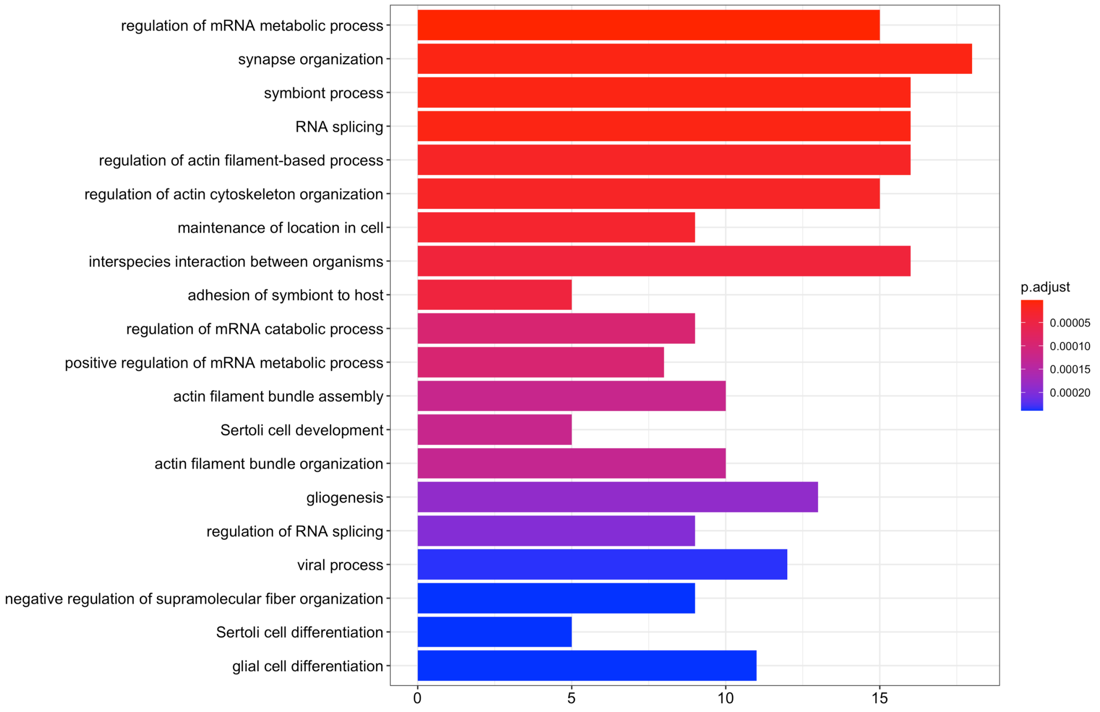

Fig. S6: Visualization of the top biological processes from the Gene Ontology database.

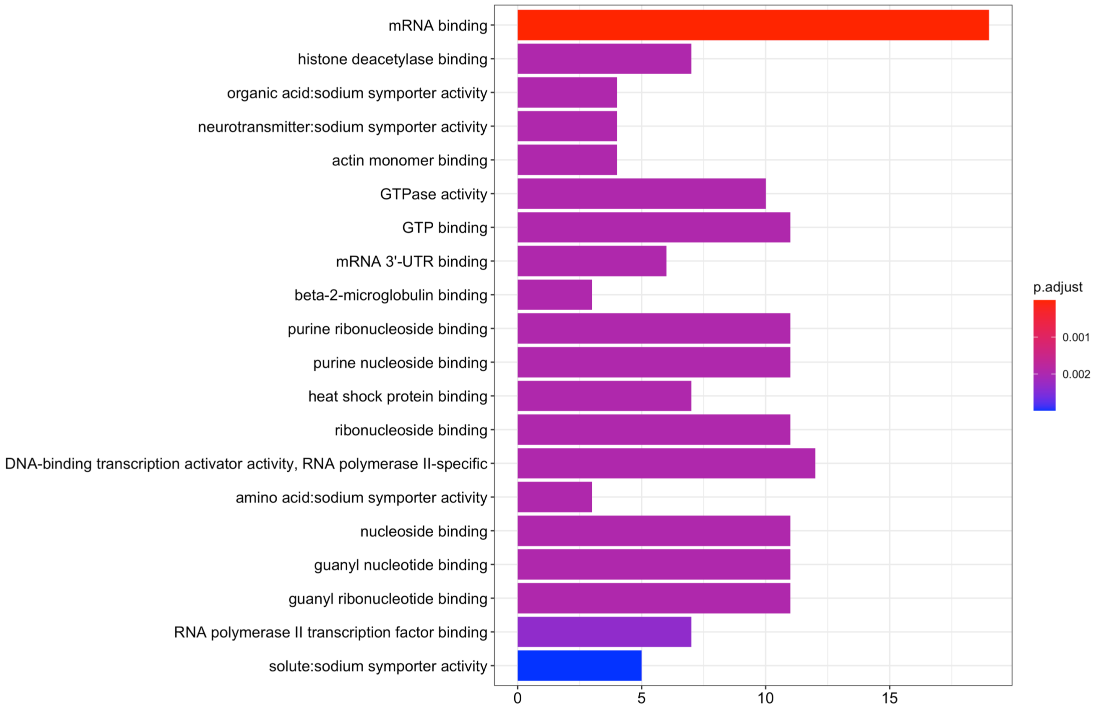

Fig. S7: Visualization of the top molecular functions from the Gene Ontology database.

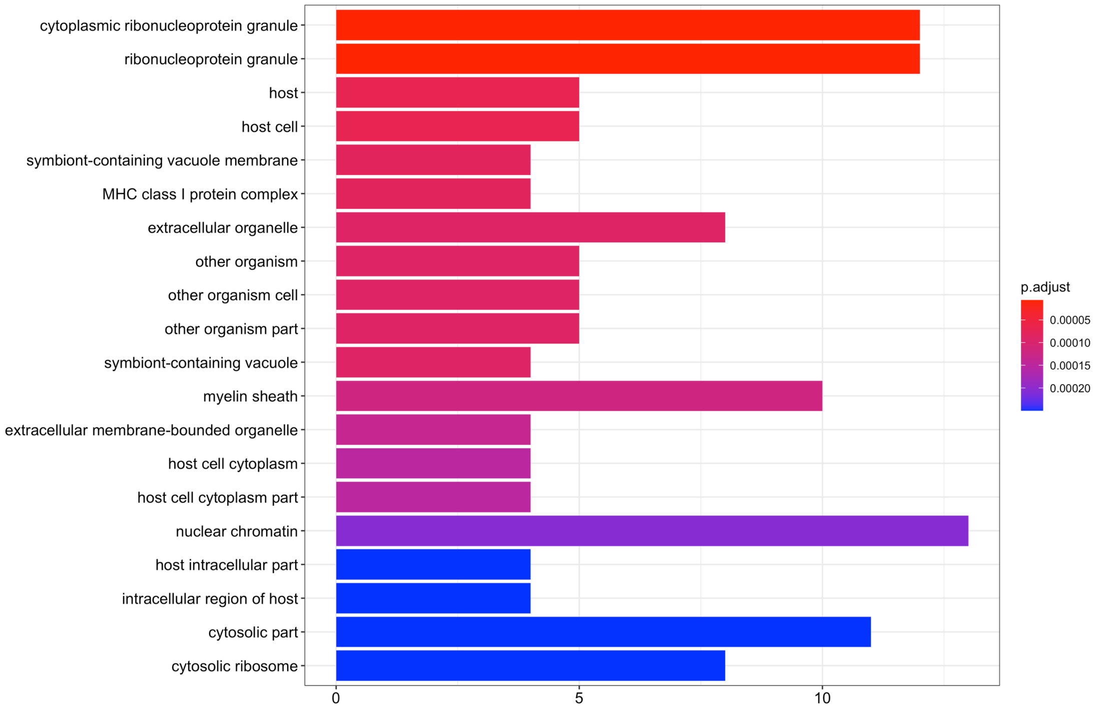

Fig. S8: Visualization of the top cellular components from the Gene Ontology database.

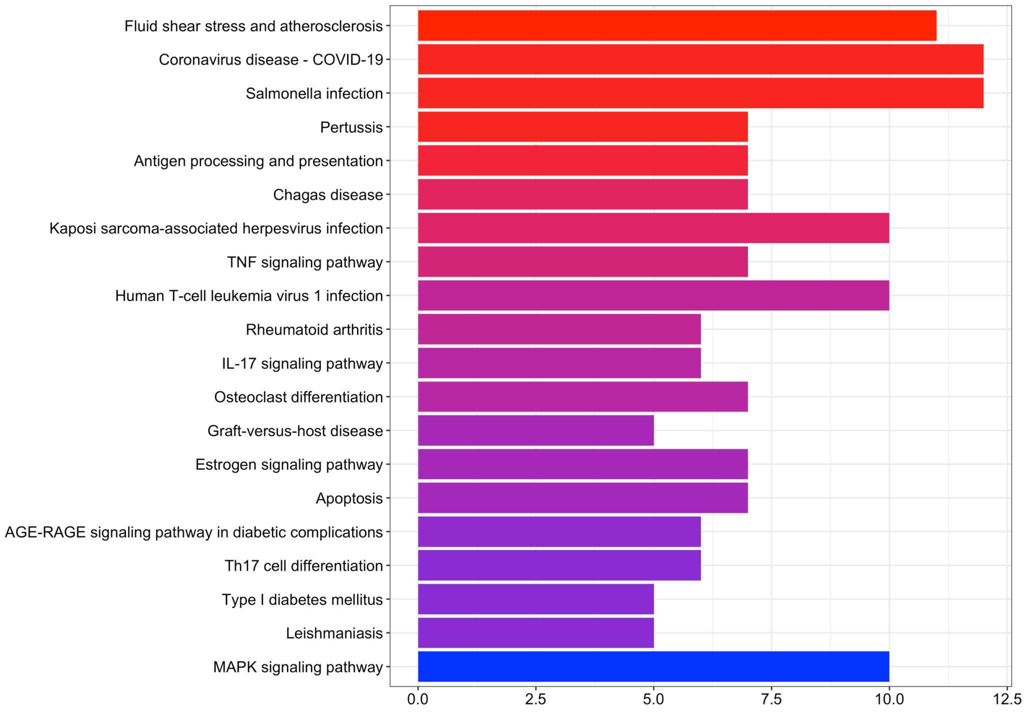

Fig. S9: Visualization of the top KEGG pathways.
